# Shifting the focus of zebrafish toward a model of the tumor microenvironment

**DOI:** 10.7554/eLife.69703

**Published:** 2022-12-20

**Authors:** Joshua M Weiss, Dianne Lumaquin-Yin, Emily Montal, Shruthy Suresh, Carl S Leonhardt, Richard M White

**Affiliations:** 1 Weill-Cornel Medical College, Tri-Institutional M.D./Ph.D. Program New York United States; 2 Memorial Sloan Kettering Cancer Center, Department of Cancer Biology & Genetics New York United States; 3 Department of Medicine, Memorial Sloan Kettering Cancer Center New York United States; Utrecht University Netherlands; Utrecht University Netherlands

**Keywords:** microenvironment, cancer, zebrafish

## Abstract

Cancer cells exist in a complex ecosystem with numerous other cell types in the tumor microenvironment (TME). The composition of this tumor/TME ecosystem will vary at each anatomic site and affects phenotypes such as initiation, metastasis, and drug resistance. A mechanistic understanding of the large number of cell-cell interactions between tumor and TME requires models that allow us to both characterize as well as genetically perturb this complexity. Zebrafish are a model system optimized for this problem, because of the large number of existing cell-type-specific drivers that can label nearly any cell in the TME. These include stromal cells, immune cells, and tissue resident normal cells. These cell-type-specific promoters/enhancers can be used to drive fluorophores to facilitate imaging and also CRISPR cassettes to facilitate perturbations. A major advantage of the zebrafish is the ease by which large numbers of TME cell types can be studied at once, within the same animal. While these features make the zebrafish well suited to investigate the TME, the model has important limitations, which we also discuss. In this review, we describe the existing toolset for studying the TME using zebrafish models of cancer and highlight unique biological insights that can be gained by leveraging this powerful resource.

## Introduction

Cancers are recognized to resemble ecosystems composed of both tumor and microenvironmental components (Joyce and Pollard, 2009). The tumor microenvironment (TME) is especially important for clinically relevant endpoints such as drug resistance and metastasis, the major causes of cancer mortality. The TME itself is composed of a highly heterogeneous collection of cells, proteins, and soluble factors that can all affect tumor phenotypes. Adding to this complexity is the long-range communication between the tumor and distant parts of the body through secreted factors such as hormones, cytokines, and extracellular vesicles (Peinado et al., 2017). This idea of cancer as a ‘whole body’ problem is not new but is difficult to study.

A major challenge is developing models that both reflect the complexity of the human TME and also facilitate its investigation. The mouse remains the mainstay for such studies, given the wide range of genetic tools, tumor models, and established protocols. The major strengths of mouse models include the close homology to human disease and the ability to express transgenes in both an inducible and tissue-specific manner (Hill et al., 2021). Despite its strengths, the mouse remains a challenging model to perturb the TME at large scale, since it often requires breeding of multiple alleles to study one or two genes in specific TME cell types. For example, modeling loss of function in the microenvironment would require generating a floxed allele in an established mouse model of cancer (i.e. BRAF;PTEN mouse model of melanoma [Dankort et al., 2009; Dhomen et al., 2009] or the KRAS;TP53 model of pancreatic cancer [Hingorani et al., 2003], etc.) and then crossing with a Cre driven by a microenvironment-specific promoter. Recent advances in mouse technologies, including ES-cell-derived ‘SpeedyMice’ (Premsrirut et al., 2011) and electroporation-based models (Leibold et al., 2020) circumvent some of these issues, but the mouse is still not optimized for large-scale experimentation given the cost of each individual animal. Additionally, high-resolution in vivo imaging in the mouse remains highly specialized (Patsialou et al., 2013) and is difficult to achieve at the single cell level. Organoid models have received increased attention due to their ability to model both intratumor heterogeneity as well as interactions with specific cell types in the TME (Neal et al., 2018) but are largely limited to studying one or two TME cell types. These limitations can make addressing specific questions related to the TME difficult or even impractical. In Table 1, we summarize the advantages and disadvantages of different cancer models in investigating the TME.

**Table 1.** Comparison of animal models in investigating the TME.

Model organism	Clinical relevance	Scale of cell-cell interactions	Ease of imaging cell-cell interactions	Drug screening throughput	Scalability of genetic perturbation
Mouse	High	High	Low	Low	Low
Organoid	High	Low	Medium	High	Medium
Zebrafish	Medium	High	High	Medium (adult)-High (embryo)	High
Flies	Low	High	High	High	High

In recent years, the zebrafish (*Danio rerio*) has evolved as a new model organism in cancer biology (White et al., 2013). Zebrafish are small (2–5 cm) tropical fish that share 70% of their genome with humans, and more than 80% of disease-associated genes are conserved (Patton et al., 2021). Its major strengths are that it is easy to genetically manipulate at large scale and is highly optimized for in vivo imaging. It is also amenable to chemical screens, allowing for identification of bioactive small molecules. Up until now, the fish has been primarily used to investigate cell-intrinsic effects of oncogenes and tumor suppressors, although it is increasingly being used to look at the TME. In its most straightforward manifestation, cell-type-specific promoter/enhancers are used to drive oncogenes, and these constructs are used to create transgenic animals that develop tumors. Although most of these cell-type-specific drivers were initially isolated for use in developmental biology studies, they were repurposed for use in these transgenic cancer models. The first clear example of this was a transgenic animal in which the T-cell-specific rag2 promoter was used to drive the MYC oncogene (Langenau et al., 2003), which resulted in T-cell leukemia in the animals. Along the same lines, the melanocyte-specific mitfa promoter was used to drive BRAF^V600E^ (the most frequently mutated gene in human melanoma) and those animals developed melanoma that closely resemble the patient disease (Patton et al., 2005). This same mitfa promoter fragment has been widely utilized in modifier screens on top of BRAF^V600E^ to drive expression of novel melanoma oncogenes such as SETDB1 (Ceol et al., 2011), SOX10 (Kaufman et al., 2016), and GDF6 (Venkatesan et al., 2018) in melanocytes. This mitfa promoter has been used to drive Cas9 to inactivate genes specifically in melanocytes, such as SPRED1, an important tumor suppressor in mucosal melanoma (Ablain et al., 2018). Beyond leukemia and melanoma, transgenic zebrafish have been used to model a wide variety of cancer types ranging from rhabdomyocarcoma to hepatocellular carcinoma, and their details can be found in other reviews (Hason and Bartůněk, 2019; McConnell et al., 2021; White et al., 2013). In addition to transgenic models, the zebrafish has also extensively been used as a model for tumor cell transplantation. This can be done at both larval/embryonic stages (prior to the development of adaptive immunity) as well as adult stages (which need to be immuncompromised due to immune rejection). This can be done using either zebrafish-derived (Heilmann et al., 2015) or human-derived cell lines (Yan et al., 2019).

**Figure 1.**
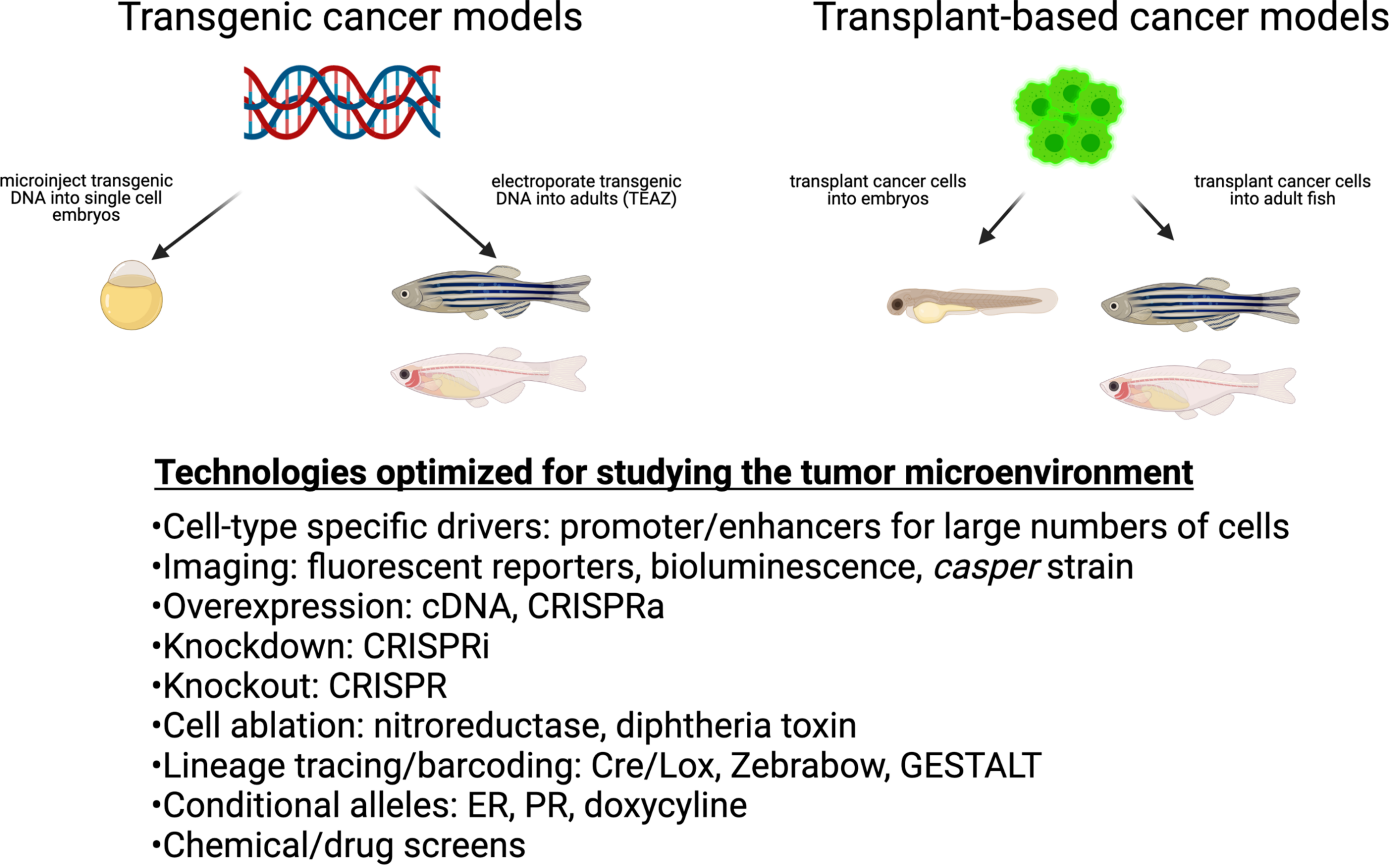
Cancer techniques in zebrafish. These include both transgenic and transplantation methodologies. While many of these techniques have been applied to the cancer cell itself, they are readily adaptable to the study of the microenvironment.

Despite the tremendous advances in cell-intrinsic modeling of cancer, relatively less attention has been paid to the TME. However, the zebrafish is well poised for such studies (Figure 1) due to its ease of genetic manipulation in a cell-type-specific manner combined with excellent in vivo imaging. Most components of the TME are conserved between zebrafish and humans, although there are important exceptions which we discuss below. In this review, we describe the technologies unique to zebrafish that will help disentangle the complexity of the TME and facilitate a deeper understanding of its role in the pathogenesis of cancer.

### Imaging the tumor/TME interaction in zebrafish

The ability to visualize how cancer cells interact with various cells in the TME is perhaps one of the major strengths of the model. Here we describe the different features that facilitate high-quality imaging in zebrafish.

### Size and numbers

The ease of high-quality imaging stems from its small size (4 mm–12 mm in embryos/larvae and ~2–5 cm in adults) and optical transparency (Singleman and Holtzman, 2014). This is especially true when studying cancer using embryonic/larval models, but many of the same principles can be applied to optically translucent adults such as *casper*, as discussed below. The smaller size makes it possible for an entire zebrafish embryo/larvae to be placed on a confocal and multi-photon microscope. The ability to image an entire animal at single-cell resolution enables investigation of not just local microenvironmental interactions but also distant ‘whole body’ interactions (Loveless et al., 2020). This is particularly important for understanding the contribution of the TME to metastasis, which is classically separated into at least four steps: intravasation from the primary site, circulation in blood stream, extravasation at distant sites, and outgrowth at those sites (Gupta and Massagué, 2006). Each of these steps is typically studied using very different model systems, since they are occurring at different scales: whereas intravasation might occur at the single-cell level (and might be studied using complex mouse intravital imaging), outgrowth represents the collection of many thousands of cells and can be studied at the whole-animal level (using methods such as bioluminescence/IVIS) (Gómez-Cuadrado et al., 2017; Hill et al., 2021). In contrast, the zebrafish can be used to image and quantify each of these steps in a single animal, since it is highly amenable to standard epifluorescence microscopy (for many cells), confocal microscopy (for single cells), and lightsheet microscopy (even now including whole embryo and adult animals). Given their small size and transparency, most questions related to cell-cell interactions can be captured using epifluorescence and confocal microscopy, although more specialized methods, such as two photon, second harmonic generation, light sheet, and micro-CT, are also possible, and their applications and technical advantages have been summarized by Loveless et al., 2020.

Beyond imaging, the smaller size of the zebrafish provides additional advantages for characterizing the TME. Spatial transcriptomics is a new RNA-sequencing technology that provides spatial resolution to gene expression and has already provided key insights into cancer-TME interactions (Longo et al., 2021). In contrast to mouse models, an entire adult zebrafish section can fit on a slide used for spatial transcriptomics thereby enabling investigation not just of local interactions but whole-body interactions. In a BRAF^V600E^ model of melanoma, spatial transcriptomics paired with single-cell RNA-sequencing (scRNA-seq) identified a distinct ‘interface’ cell state in both tumor and TME cells where they are in physical contact with one another. This cell state is associated with upregulation of cilia genes, and its function is an area of investigation (Hunter et al., 2021).

In addition to spatial transcriptomics, the smaller size of the zebrafish makes it feasible to work with hundreds or thousands of animals for an experiment and increases the statistical rigor in a way that is not feasible in mice. Zebrafish embryos can be placed in a 96-well plate for chemical screening (Patton et al., 2021), and 1 liter of space can house many more fish than mice. A single breeding pair can yield hundreds of embryos in contrast to approximately a dozen offspring in mice. Therefore, a major advantage of doing these studies in zebrafish, as opposed to mice, comes down to numbers: many different donor cells can be used, and many different genes or drugs can be tested (either as a candidate approach or in a screening approach) (Fazio et al., 2020).

### Optical transparency

Most zebrafish imaging has traditionally been done during the embryonic and larval stages, since those young animals are naturally transparent. A natural use of the fish at this stage is for cell transplantation. In the larval xenograft assay, fluorescently labeled cancer cells are transplanted into the circulation of the developing animal. If cells in the TME are labeled in a different color, then a very high-resolution view of tumor/TME interaction can be obtained with this assay. Numerous examples using this assay exist that have yielded both mechanistic and clinically relevant information. For example, the human RhoC gene was engineered into a variety of human cancer cell lines and then transplanted into fli1a-GFP (green fluorescent protein) transgenics, which revealed that these cells ‘open up’ the vasculature via secretion of VEGF, which ultimately allows them to invade into new tissues (Hoeppner et al., 2015). This larval xenograft assay can also be used to study metastasis and drug response, such as the anti-VEGF molecule bevacizumab using PDX (patient-derived xenograft) models (Rebelo de Almeida et al., 2020). A xenograft of melanoma cells co-cultured with lymphatic endothelial cells revealed an increase in distant cancer cell dissemination in zebrafish (Pekkonen et al., 2018). By coupling this assay with cell ablation approaches, the role of certain cell types such as macrophages has been shown to be required for tumor angiogenesis, a key component of tumor growth (Britto et al., 2018). This model has recently been extended into patient ‘avatars’ to investigate responses to specific drugs in vivo (Fazio et al., 2020; Fior et al., 2017). Using two matched patient-derived colorectal cancer cell lines from the primary tumor as well as a lymph node metastasis in a larval xenograft assay, a recent study revealed how cancer cells from the same patient generate different TMEs through differential recruitment of neutrophils and macrophages, resulting in opposing implantation kinetics. Subsequent scRNA-seq indicated IFN/Notch vs IL10 signaling in mediating subclonal selection of escaper cells, pointing to a role for innate immunity (Póvoa et al., 2021). Numerous other examples of using this larval xenograft assay exist, and there are some major advantages to this assay compared to mouse xenografts (Pascoal et al., 2020). The assay is very rapid, can usually be readout in 1–5 days post-transplant, and is easily amenable to single-cell imaging. In addition to transplant assays, transgenic cancer models have also been used at this early developmental timepoint to investigate the direct interaction of innate immune cells with cancer cells and mechanisms of pro-tumoral growth. Important discoveries from these models include the tumorigenic consequences of the wound-associated recruitment of neutrophils, the ability of innate immune cells to promote tumor invasion through proteolytic breaching of the basement membrane, and the pro-tumoral effects of leukocyte-derived PGE2 (Antonio et al., 2015; Feng et al., 2012; Feng et al., 2010; van den Berg et al., 2019).

While these embryonic/larval assays are useful, they are not without limitations. In particular, adaptive immunity (i.e. T and B cells) as well as stromal cells are key components of the TME that are not present in embryos and arise later in adulthood (Minchin and Rawls, 2017; Page et al., 2013; Trede et al., 2004). Recognizing that these studies would be enhanced if they could be done in adult animals, we previously developed a more optically transparent strain of zebrafish called *casper* that is amenable to adult imaging (White et al., 2008). We initially used this line for allograft experiments, in which the donor tumor cells were derived from a transgenic zebrafish (i.e. ZMEL1-GFP cells), which allowed us to identify a requirement for endothelin (EDN3) signaling from the TME in melanoma cell dissemination (Kim et al., 2017). However, these adult fish still have limitations as well, since they still need to be irradiated to eliminate adaptive immunity. Nonetheless, they can be highly useful for studying cancer cells in an adult animal. Toward that end, adult immunocompromised *casper* fish have now been generated, such that xenograft studies using human cancer cell lines or PDXs can also be performed (Yan et al., 2019). This has been used for drug studies, revealing the efficacy of PARP inhibitors and DNA-damaging agents in rhabdomyosarcoma, and responses to immune therapies as well. We are now also using *casper* for transgenic approaches such as transgene electroporation into adult zebrafish (TEAZ; discussed below), allowing for better evaluation of both tumor and TME with intact immune systems and other components of the TME. While it is clear that adult imaging is not as high-throughput as embryonic/larval imaging, it is still entirely feasible for most zebrafish labs to accomplish this without sophisticated equipment.

### Continuous live imaging

The combination of small size and optical transparency also enables continuous live imaging. In mice, high-resolution live imaging typically requires sacrificing the animal or utilizing highly specialized techniques that are often invasive (Stoletov et al., 2007). In contrast, zebrafish embryos/larvae in particular are ideal for imaging cell-cell interactions for hours or even days. The power of continuous imaging is highlighted by a recent study showing metastatic tumor cell clusters composed of divergent cell states cooperating with one another to promote melanoma metastasis. By isolating cell lines enriched for one state vs another and transplanting them into embryos, continuous confocal imaging demonstrated extravasation of circulating tumor cell clusters with invasive cells on the leading edge and proliferative cells following. Continuous high-resolution imaging in combination with facile transgenesis was essential to identify the transcription factor TFAP2 as the major regulator of these divergent cell states (Campbell et al., 2021). Similarly, live imaging of xenografts in zebrafish embryos was used to investigate the role of hemodynamic flow forces in the arrest, adhesion, and extravasation of circulating tumor cells finding that low flow vessels favor tumor cell arrest, but high flow favors endothelial remodeling that permits efficient extravasation (Follain et al., 2018).

### A toolbox of cell-type-specific drivers in zebrafish that can be used to study the TME

Broadly speaking, any cell type within the body can be considered to be part of the TME, depending on the exact tumor type. For example, in melanoma, the tumors largely arise in melanocyte precursors, which are surrounded by the normal resident cells of the skin – keratinocytes, fibroblasts, and adipocytes (Golan et al., 2015; Kaur et al., 2016; Zhang et al., 2018). These seemingly normal resident cells all play roles in shaping tumor phenotypes such as proliferation and invasion. The crosstalk between TME cells and tumor cells is likely to be bidirectional, in that each cell will undergo changes when next to each other. For example, it is now well understood that normal fibroblasts take on new transcriptional characteristics to become ‘cancer-associated fibroblasts’ (Sahai et al., 2020); similarly, ‘tumor-associated macrophages’ can be altered in the presence of a tumor to take on either pro or anti-tumorigenic properties (Quail and Joyce, 2017). One still unresolved question is how this reprogramming occurs. Soluble factors like TGF-beta from stromal cells can enact broad transcriptional rewiring of recipient tumor cells, but there are likely other metabolic or cell-cell mechanisms that epigenetically rewire the tumor cell as well. For example, LIF is known to enact epigenetic changes to convert fibroblasts into CAFs (cancer-associated fibroblasts) (Albrengues et al., 2015), and pancreatic stellate cells can be reprogrammed by Vitamin D (Sherman et al., 2014). Recent advances in scRNA-seq, ATAC-seq, and spatial transcriptomics will continue to add nuance to these studies, since it will become clearer how both the TME and the tumor get altered over time and space. The general importance of resident cell types, whether normal or altered by the tumor cells, opens up the possibility of studying and perturbing them in much greater detail, which offers many new therapeutic opportunities.

**Figure 2.**
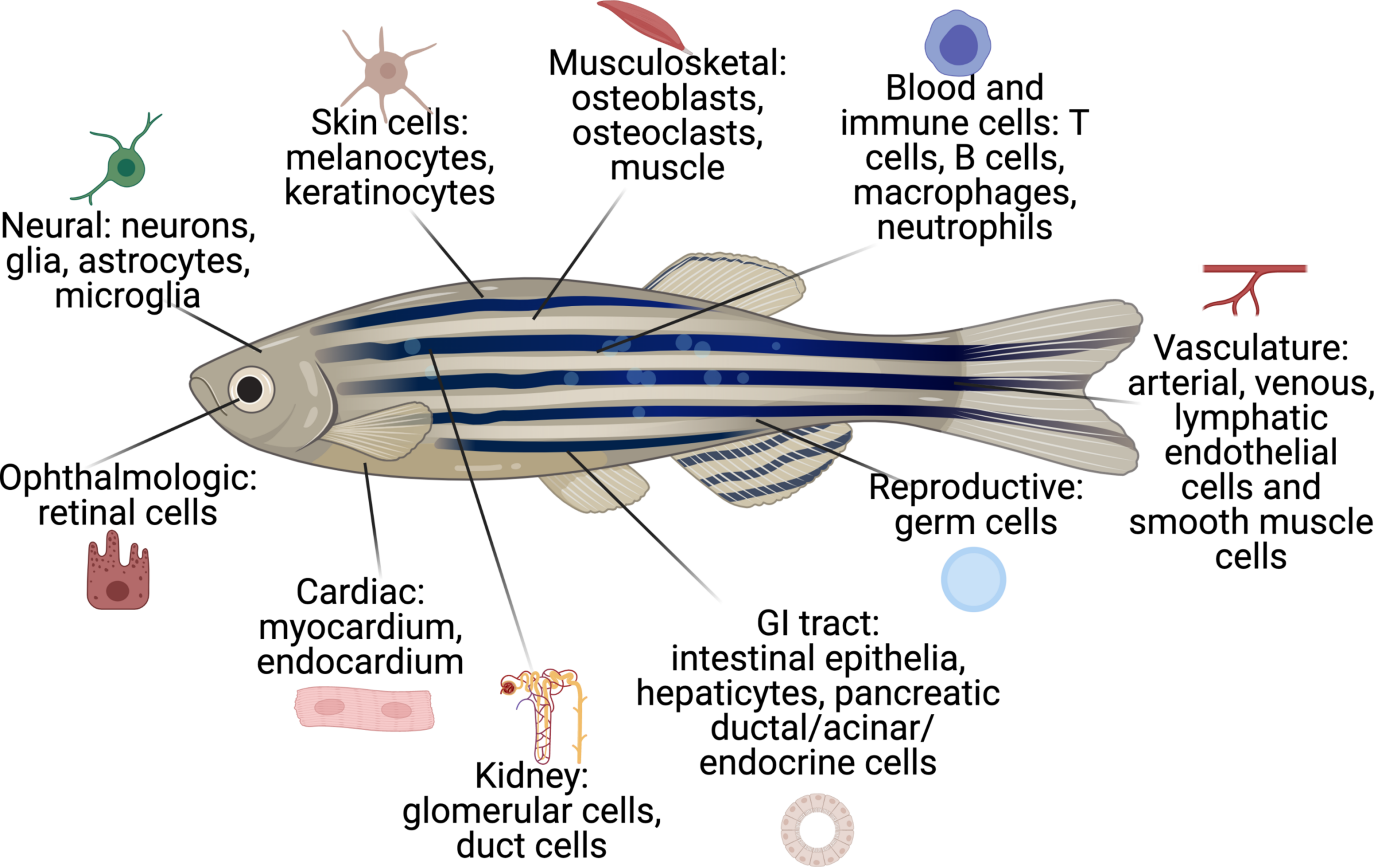
The cell types that have existing transgenic reporters in zebrafish. The specific promoter/enhancers driving expression in each of these cell types are shown in Supplementary file 1.

**Figure 3.**
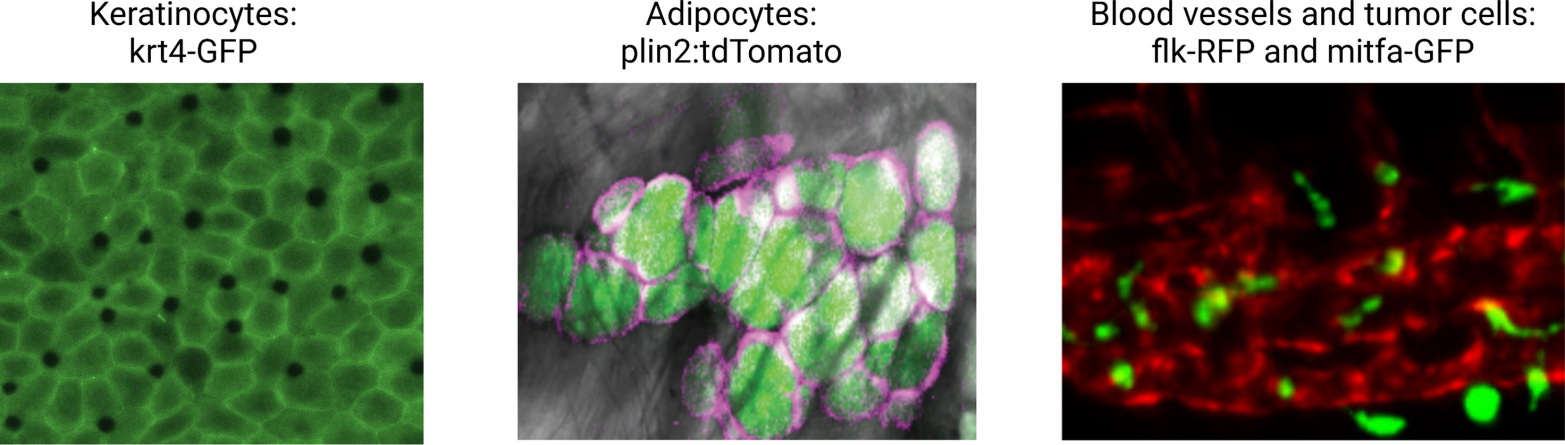
Illustrative examples of transgenic zebrafish reporters for specific cell types relevant to the microenvironment. Examples shown include the keratinocytes (marked by krt4-GFP), lipid droplets, and adipocytes (marked by a plin2:tdTomato fusion), and the blood vessel/melanoma interface (marked by the flk1-RFP and mitfa-GFP reporter, respectively). See Supplementary file 1 for a full list of references for these and other lines. Second panel is reproduced from Figure 1D in Lumaquin et al., 2021.

In order to facilitate the study of these diverse cell types, we have developed a comprehensive database of cell-type-specific promoters or enhancers that have been published by the zebrafish community (Figure 2 and Supplementary file 1). Cell-type-specific promoter/enhancers allow for labeling of the cell type of interest (by using the promoter to drive expression of a fluorophore), or for manipulation of gene expression within the desired cell population (by expressing cell-type-specific Cas9, cDNAs, or ablation cassettes). A few examples of TME cells readily labeled in zebrafish transgenics are shown in Figure 3. Most of these have been studied during embryogenesis, and whether all of them will faithfully work in adults is still unknown, although anecdotal experience (i.e. *mitfa, krt4, fli1a*, etc.) shows this to be the case. Although comprehensive compendium of mutant zebrafish strains, expression patterns, and reagents are already available on open source platforms, such as ZFIN (https://zfin.org), there is currently no such list of promoters/enhancers. We are proposing to use this resource and directly apply it to a comprehensive in vivo analysis of the TME.

The majority of these cell-type-specific promoter/enhancers were originally studied in the context of developmental biology. For example, as mentioned above, a promoter fragment of the mitfa gene, which is specific to melanocytes, was used to drive GFP, allowing for visualization of when and where melanocytes are specified during embryogenesis (Lister et al., 1999). Many other skin promoters have been identified: *krt4* for superficial keratinocytes (Ju et al., 1999), *tp63* for basal keratinocytes (Eisenhoffer et al., 2017; Lee et al., 2014), and *plin2* for adipocytes (Zhang et al., 2018). The blood system has many promoters that allow for imaging and isolation of specific cell types: *cd79* for B-cells (Liu et al., 2017), *lck/rag2* for T-cells (Jessen et al., 2001; Langenau et al., 2004; Langenau and Zon, 2005), and *mpeg1* for macrophages (Ellett et al., 2011). These transgenically marked cells can be isolated and manipulated in the same way that in mammalian systems cocktails of antibodies can be used in flow cytometry to isolate those same cells. A broadly useful promoter has been *fli1a*, which marks the endothelial cells lining blood vessels and has been widely used in many developmental and regenerative studies due to its high specificity and strong expression (Lawson and Weinstein, 2002). The nervous system is another area that has seen great development of such promoters. Multiple neuronal subtypes have been appropriately marked by promoters such as *gsx1* (general neuronal), *vglut2a* (glutaminergic neurons), gad1b (GABAergic neurons), among many others (Satou et al., 2013). The list of such promoters continues to grow and includes cells from virtually every organ, including the GI tract, the liver, the eye, the endocrine system, reproductive organs, and the heart. However, while the database features an impressive set of cell-type-specific promoters, certain cell-type-specific promoters are so far missing, including for natural killer cells, implicated as antitumorigenic seemingly a major contributor of controlling metastatic dissemination (Chan et al., 2020; Ilander et al., 2017; Lakshmikanth et al., 2009).

Our ability to identify new promoter/enhancers for even rare cell types or cell states will continue to increase. scRNA-Seq has provided a tremendous amount of insight into not only distinct cell ‘types’ but also cell ‘states’, which are marked by distinct transcriptional programs (Farrell et al., 2018). In both development and cancer, some of these cell types or states can be rare, making them very difficult to study in vivo. This data from the scRNA-seq can be used to select genes whose expression is likely to be representative of that particular cell type or cell state of interest. This can be tested by cloning promoter fragments for that gene and then determining whether it faithfully marks the cell state of interest. However, despite the tried and true method of cloning promoter or enhancer fragments to drive transgenic cassettes, or using BAC-transgenic fragments (Suster et al., 2011), advances in CRISPR genome editing are likely to eventually replace this method by directly knocking in transgenic cassettes of interest at the endogenous locus (Auer et al., 2014; Welker et al., 2021; Wierson et al., 2020). Beyond the known promoter/enhancer regions cloned by the zebrafish community, a recent exciting advance comes from the international DANIO-CODE consortium (Baranasic et al., 2022), which has comprehensively cataloged ~140,000 cis-regulatory regions in zebrafish development, many of which will mark specific cell types and states. Another interesting resource to expand this repertoire is random insertional screens (Balciunas et al., 2004). In this case, transposable elements containing basal promoters driving GFP or a Gal4-UAS construct are randomly inserted into the genome, and then fish with potentially interesting GFP expression patterns are isolated and the insertion site sequenced (Abe et al., 2011). Such an approach can lead to identification of cell types having unexpected biological specificity. For example, a recent study using this approach identified a neuronal subpopulation of cells essential for fear conditioning (Lal et al., 2018). It is not hard to imagine using this screen to find promoters for rare cell types or cell states, as long as one knows what they are looking for.

While most cell types are conserved from zebrafish to humans, there are notable exceptions, such as gills vs lungs, lymphatics without lymph nodes, and hematopoietic stem cells (HSC) contained within the kidneys (referred to as kidney marrow) as opposed to bone marrow. Because lung, lymph node, and bone marrow metastases are common metastatic sites in human cancer (Chaffer and Weinberg, 2011), this is an important limitation of the zebrafish, and investigating the TME interactions for these particular metastatic sites may be better suited in other models. Despite these differences, the zebrafish equivalent of these structures still has the potential to provide immense insight into tumor-TME interactions. The gills like lungs are sites of oxygen exchange and contain many of the same cell types, including alveolar epithelial type I cells (called pavement cells in fish), goblet cells, and neuroepithelial cells (Cadiz and Jonz, 2020). Both lungs and gills are also important sites of barrier immunity where zebrafish have been used to study the developmental origins of tissue-resident macrophages (Lin et al., 2020) and also gill/lung regeneration (Cadiz and Jonz, 2020). Furthermore, the zebrafish has become a well-established model to investigate lymphangiogenesis and the HSC niche (Flores et al., 2010; Wattrus and Zon, 2018). Pharmacogenetic screens in zebrafish have identified inhibitors of tumor lymphangiogenesis (Astin et al., 2014), and embryo transplant assays have been used to model bone marrow metastasis (Paul et al., 2019; Sacco et al., 2016). Furthermore, zebrafish have a brain and liver and therefore investigating the tumor-TME interactions at these other common metastatic sites are well suited for modeling in zebrafish.

### Genetically perturbing the TME

While imaging-based approaches are important, the true power of the zebrafish will be brought to bear via perturbation experiments. To really move this approach forward, we must alter these genes not only in tumor cells, but in specific TME cell types as well, which is where the available cell-type-specific promoters become important. This translates to at least three different approaches – overexpression of specific genes, knockout/knockdown of specific genes, or ablation of the entire cell type of interest. For overexpression, the most straightforward way to do this is via cDNA cassettes. For example, increased expression of a growth factor such as endothelin under a keratinocyte-specific promoter like *krt4* or *tp63* would be expected to promote the growth of melanoma cells, as has been shown in mouse studies (Benaduce et al., 2014). For knockout or knockdown, CRISPR is clearly the technology of choice. Competing technologies such as TALENs or ZFNs are too cumbersome for routine use, and RNAi approaches have generally failed in zebrafish. In contrast, CRISPR has been widely adopted by the zebrafish community with great success (Hwang et al., 2013). Although initially used mainly during development to achieve global knockout (Burger et al., 2016) (by injecting Cas9/sgRNAs into single cell embryos), the recent development of tissue-specific CRISPR plasmids allows for cell-type-specific in vivo gene editing (Ablain and Zon, 2016). These plasmids allow incorporation of diverse cell-type-specific promoters to drive Cas9 on one side of the vector, while the other side of the vector contains a U6 promoter driving an sgRNA of interest. This was first applied by using the *gata1* promoter to drive Cas9 (Ablain et al., 2015), which drives expression in red blood cells, to knockout the gene urod, known to be important in heme synthesis. By injecting this into single-cell embryos, they were able to recapitulate the phenotype of a known global mutant of urod, but now in a cell-type-specific manner. Similarly, the MASERATI plasmid has been used to knockout genes specifically in melanocytes to generate melanomas (Ablain et al., 2018). Another transient approach based on the injection of chemically modified synthetic gRNAs into embryos of transgenic fish expressing Cas9 in a lineage-specific matter based on the GAL4/UAS system showed promising results in gene editing of zebrafish neutrophils and macrophages and could be in principle extended to other cell types of interest (Isiaku et al., 2021). With many newer CRISPR systems now available one can perform unique genetic edits in different TME cell types simultaneously (Liu et al., 2019). Furthermore, greater cell type specificity can be achieved with anti-CRISPR, which can protect cell types from unwanted genetic editing (Nakamura et al., 2019). CRISPR could also be exploited for overexpression and silencing (rather than knockout) using CRISPRa or CRISPRi (Gilbert et al., 2014), although this has minimally been tested in fish so far. Finally, entire TME cell types can also be ablated using a wide variety of ablation cassettes driven by these same cell-type-specific TME promoters. Many such systems have been tested in fish including the nitroreductase (Curado et al., 2008; Pisharath et al., 2007) and diphtheria toxin (Wan et al., 2006) systems.

The zebrafish also offers a diverse set of genetic tools that enable conditional and inducible overexpression, CRISPR editing, and cell ablation, similar to those established in mice. For conditional overexpression, an estrogen receptor variant (ERT2) has been fused to the GAL4 transcriptional activator. By driving the ERT2-GAL4 under the control of a TME-specific promoter and a cDNA under a UAS promoter, one can drive overexpression of a gene of interest in both a cell-type-specific and temporally controlled manner through the addition of tamoxifen (Akerberg et al., 2014). A similar system using a GAL4 ecdysone receptor fusion (GAL4-EcR) has also been reported (Esengil et al., 2007). Other conditional overexpression systems successfully utilized in zebrafish include the mifepristone-inducible LexPR system (Emelyanov and Parinov, 2008) as well as TET-inducible systems (Knopf et al., 2010). Although these inducible systems have been reported, they are unfortunately not as reliable as they are in mouse transgenics and therefore are not as commonly used in zebrafish models. For knockout studies, Cre-Controlled CRISPR (3 C) has been reported whereby expression of Cas9 requires removal of a lox-stop-lox cassette under the regulation of a tissue-specific CreERT2, thereby making CRISPR editing both spatially and temporally controlled. In addition to controlling Cre activity with tamoxifen, the authors in this study also used a heat inducible promoter hsp70l to induce the expression of Cas9, further increasing the temporal control and specificity of editing (Hans et al., 2021). Compared to mouse models, the utilization of temporal/inducible genetic tools is still a work in progress. However, given the ease of genetic manipulation and the abundance of TME-specific promoters, the zebrafish is well suited for investigating the role of the TME in cancer progression.

### Technologies to make it scalable and predictable: non-germline approaches

This confluence of many different cancer models with many different TME perturbations opens exciting opportunities to dive deeply into these interactions. However, a major goal of doing this in zebrafish, as opposed to mice, is that we can generate much higher throughput and scalability. As alluded to above, knocking out one to two genes in the TME is important, but in an ideal world, we would like to be able to knockout many genes – perhaps dozens or hundreds – in the TME very rapidly. A roadblock to doing this is germline transmission. In traditional genetic alleles, the perturbation of interest is passed through the germline to generate so-called ‘stable’ lines. While this germline approach is routinely applied for a focused study of a small number of genes, scaling up to a larger number of genes would involve time-consuming breeding of animals over multiple generations, which is not really feasible. This challenge has been addressed by advances in non-germline (i.e. somatic) genetic approaches in the zebrafish. Again taking a page from developmental biology, the initial non-germline approaches involved F0 or ‘crispant’ approaches. In this case, ribonucleoprotein complexes composed of recombinant Cas9 plus synthetic sgRNAs were complexed together, injected into single-cell zebrafish embryos, and the resultant F0 animals were directly screened for phenotypes of interest (Burger et al., 2016). More sgRNAs per gene have been found to achieve a phenotype of more than 90% in a study investigating cardiomyocyte development (Wu et al., 2018). The MASERATI system described above uses the same logic – mosaic expression of the plasmid leads to knockout in some but not all cells, but is sufficient to drive a phenotype. In the cancer setting, such non-germline, mosaic F0 approaches have been very important in identifying oncogenes (i.e. SETDB1 and CRKL) (Ceol et al., 2011; Weiss et al., 2022) and tumor suppressors (i.e. SPRED1) (Ablain et al., 2018) and can be used to test effects in thousands of animals per experiment, a major benefit of the zebrafish system. Beyond the ability to quickly generate a large number of transgenic animals, this approach also enables the generation of genetically complex models with simultaneous integration and expression of overexpression cassettes, CRISPR systems, and fluorescent reports. For example, in a study investigating a rare subtype of melanoma, called acral melanoma, the authors used up to seven genetic constructs simultaneously (Weiss et al., 2022), providing enormous flexibility in investigating the role of a given gene.

In a typical mosaic F0 experiment, the plasmid is injected into a single-cell embryo, and then one waits several months for the animals to grow up for the tumors to stochastically emerge over a long period of time. There is no way to know when each individual animal will develop a tumor, which can range from weeks to years. Moreover, because the plasmid is mosaically expressed, it is impossible to easily track metastasis: the appearance of many tumors in one fish could be due to either multiple primary tumors, or alternatively a single tumor that then metastasized to new locations. To address this, but still maintain the advantages of non-germline transmission, our lab recently developed an electroporation-based approach called TEAZ (Callahan et al., 2018). This approach bypasses the single-cell embryo injection entirely, and instead microinjects the plasmids of interest into the adult tissue of interest followed by a brief electroporation. This system generates melanomas under the *mitfa* promoter using essentially the same plasmids described above (Callahan et al., 2018). The fish develop these tumors precisely at the electroporation spot and nowhere else, making it both predictable and amenable to tracking progression and metastasis. We recently demonstrated the utility of TEAZ for metastasis in a screen in which we tested genes gleaned from human transcriptomic datasets (Suresh et al., 2022). This showed a specific role for the cholesterol transporter GRAMD1B as a bonafide metastasis suppressor. Because these non-germline alleles could be generated so quickly, this further demonstrates the utility of zebrafish for such screens.

Bypassing germline transmission solves a major problem (time) but creates a whole new set of challenges. Because each individual animal expresses the plasmids differently than the next animal, the onset of phenotypes will vary widely. This can largely be overcome by using a large enough cohort such that any ‘signal’ outweighs the ‘noise’ in terms of phenotype. Another issue is copy number. When we express plasmids mosaically, we have little control over how many copies integrate into the genome, where they integrate, and how well these copies are expressed. The development of ‘landing spots’ such as that developed using the AAVS1 locus in ES cells (González et al., 2014) or attP/aTTB system in *Drosophila* (Knapp et al., 2015) and explored in zebrafish (Mosimann et al., 2013) would help this issue tremendously.

But perhaps the biggest challenge in these non-germline approaches will be efficiency of manipulating the TME cells. When we manipulate the tumor cell itself by manipulating oncogenes or tumor suppressor genes, efficiency is not a major issue because Darwinian selection will allow any clone with even a slight evolutionary advantage to take over and grow out. Thus, even a single cell with the right combination of genetic alterations (i.e. a cDNA for BRAF^V600E^ and an sgRNA against *p53*) will expand massively over time. But this is not true for cells in the TME. When we use mosaic embryo injections or TEAZ to label or manipulate a TME cell type, there is no particular advantage to that cell. Thus, the number of cells that are initially labeled is of paramount importance. If we can solve the efficiency problem, an ongoing challenge, it is not hard to envision that we could now use mosaic embryo injections or TEAZ to perturb the TME using specific promoters for each cell type, along with cDNA or CRISPR approaches.

### Capturing the evolutionary history of the TME

The challenge of unraveling the multifaceted relationships of the TME in part stems from the bidirectionality of cell-cell communication and the interdependency of multiple cell types or cell states in establishing a particular tumor phenotype. Numerous studies have demonstrated how cancer cells can reprogram neighboring fibroblasts (cancer-associated fibroblasts) or macrophages (tumor-associated macrophages), and that these cells in turn can promote tumor proliferation, invasion, and metastasis (Pathria et al., 2019; Sahai et al., 2020). In order to prove the reprograming of these particular TME components, lineage tracing experiments are critical. In mice, this has traditionally been performed using Cre-lox systems, controlling a fluorophore with the Cre under the control of a tissue-specific promoter (Wagner and Klein, 2020). This has been expanded to include multiple combinations of fluorophores, known as Brainbow, for increased cell resolution (Livet et al., 2007). The zebrafish has similar technologies available, such as the GFP-mCherry ‘Switch fish’ (Mosimann et al., 2011) or ‘zebrabow’, which have a few or multiple fluorophores under the control of a tissue-specific Cre (Pan et al., 2013).

While these technologies are helpful for establishing developmental and lineage relationships among a few or dozens of cells, they are not capable of doing so for an entire organism. Recent technologies such as scGESTALT and LINNAEUS utilize the combination of scRNA-seq and CRISPR editing to simultaneously increase the resolution of barcoding to thousands of cells as well as transcriptionally profiling their cell state along a particular developmental lineage (Wagner and Klein, 2020). scGESTALT has the advantage of capturing multiple developmental timepoints, which improves the temporal nature of establishing lineage relationships (Raj et al., 2018). Both scGESTALT and LINNAEUS were first implemented in zebrafish given the ease of genetic manipulation (Raj et al., 2018; Spanjaard et al., 2018). One can envision utilizing these technologies to systematically profile the reprogramming of all the components of the TME (not just one or two cell types) through multiple timepoints in tumor progression.

**Figure 4.**
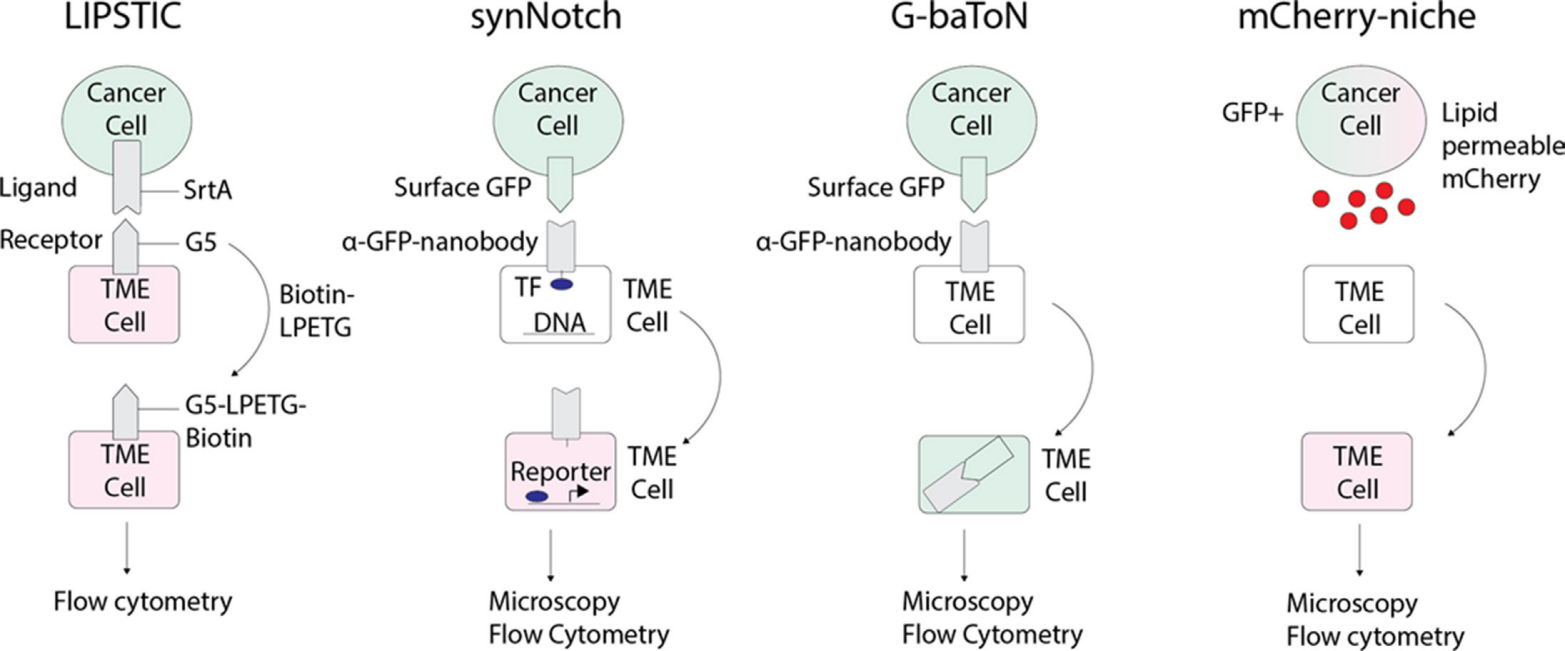
Emerging technologies that can be applied to track tumor/tumor microenvironment (TME) cell communication in zebrafish. LIPSTIC (Labeling Immune Partnerships by SorTagging Intercellular Contacts) utilizes the *Staphylococcus aureus* transpeptidase sortase A (SrtA) that can transfer peptides with LPXTG motifs to proteins containing five N-terminal glycine residues (G5) (Pasqual et al., 2018). By fusing SrtA to a ligand in a donor cell type and G5 to the cognate receptor of a receiver cell type, addition of LPETG marks the recipient G5-expressing cell. synNotch (synthetic Notch) is a versatile system in which a donor cell expresses a ligand or antigen, such as surface GFP, and the receiver cell expresses a chimeric receptor consisting of an extracellular sensing module such as an anti-GFP nanobody, a Notch core, and an intracellular transcription factor (Morsut et al., 2016). When the sensing module of the receiver cell is activated by interaction with the donor cell, the Notch core is cleaved leading to release of a transcription factor, such as Gal which can then activate downstream UAS reporters. G-baToN (GFP-based Touching Nexus) similarly utilizes surface GFP expression on a donor cell and anti-GFP nanobody expression on a receiver cell (Tang et al., 2020). Interaction of surface GFP with the anti-GFP nanobody leads to endocytosis and transfer of the GFP from the donor cell to the receiver cell, allowing for downstream analysis. mCherry-niche overexpresses a soluble and lipid permeable form of mCherry in donor cells, which is then acquired by neighboring receiver cells (Ombrato et al., 2019). By expressing mCherry-niche in GFP+ cancer cells, they can be distinguished from GFP - microenvironment cells that receive mCherry.

In addition to elucidating the lineage relationships of various TME components with barcoding, characterizing the functional consequences of heterotypic interactions in a systemic and unbiased manner is critical for elucidating the role of the TME in tumor progression. These newer cell tracing techniques (Figure 4) include Labeling Immune Partnerships by SorTagging Intercellular Contacts (LIPSTIC; Pasqual et al., 2018), SynNotch (Toda et al., 2018), mCherry-Niche (Ombrato et al., 2019), or G-baToN (Tang et al., 2020). SynNotch involves using a modified Notch ligand expressed and receptor that activates expression of a gene of interest after the receptor-expressing cell population has a direct cell-cell interaction with the ligand-expressing cell population. This technique has been used to improve the specificity and activity of CAR T cells in solid tumors (Hou et al., 2021). It has also been utilized to target Wnt activity in a model of zebrafish hepatocellular carcinoma (Wang et al., 2021). LIPSTIC utilizes a bacterial sortase-A that can tag molecularly tag predefined cell-cell interactions of interest and can be analyzed via flow cytometry ex vivo (Pasqual et al., 2018). mCherry-Niche is a labeling strategy whereby cancer cells overexpress a cell permeable mCherry fluorophore which is then taken up by neighboring TME cells that can be characterized in an unbiased manner (Ombrato et al., 2019). Lastly, G-baToN similarly relies on nanobody-directed fluorescent protein transfer to identify heterotypic cell-cell interactions (Tang et al., 2020). Although these were developed in mice, in principle they could be adapted for use in zebrafish. These technologies offer the exciting prospect of identifying unexpected cell-cell interactions. Given the unique imaging capabilities of the zebrafish, it can serve as a model to investigate the nature of a particular cell-cell interaction across space and time and then simultaneously use CRISPR or overexpression cassettes to functionally perturb these interactions.

### Future applications, and limits, of studying the TME in zebrafish

Our understanding of the ways in the TME can influence tumor phenotypes is continuing to expand. The explosion of interest in immunotherapy, a very TME-centered approach to cancer, has led to new and exciting explorations of the ways in which targeting of the TME can have meaningful effects for patients. Below, we highlight several areas in which the zebrafish models are especially well suited to help address fundamental questions in tumor/TME biology and discuss the limitations using this model.

### Heterotypic interactions between tumor cells and TME cells

One implication of the tumor/TME pairing is that heterotypic cell-cell interactions between tumor and TME cells may contribute to some tumorigenic phenotypes. This can occur via secreted factors or via direct cell-cell contact. The precedent for this is in part again provided by the developmental biology literature. Contacting cells can form cell-cell junctions which play critical roles in many aspects of development (i.e. axis elongation, gastrulation, and limb budding). Cells can form these contacts through multiple mechanisms including adherens junctions which are formed and remodeled during development (Cavey and Lecuit, 2009) and can regulate cell fate specification (Barone et al., 2017). In addition, cell-cell contact is needed to maintain intrinsic directionality of cell migration as seen in migrating prechordal plate cells during gastrulation (Dumortier et al., 2012). It is clear that cancer cells co-opt and utilize many of these same mechanisms when tumor cells meet TME cells. In glioblastoma cell lines, contact with mesenchymal stem cells causes increased expression of proteases that enhances cancer cell invasion (Breznik et al., 2017). Metastatic human colorectal cancer cells home to the perivascular niche where endothelial cells can secrete factors promoting the cancer stem cell phenotype (Lu et al., 2013). Metastatic melanoma cells that invade within the subcutaneous tissue are adjacent to adipocytes which can directly transfer fatty acids to melanoma cells and promote proliferation and invasion (Zhang et al., 2018). The embryonic/larval xenotransplant approach, or adult xenotransplant approach in immune-compromised fish, can be used to study these types of interactions at high resolution and high scale. However, one major disadvantage of these transplant-based studies is that the animals do not have a fully intact immune system, which is critical since these immune cells are central to tumor biology. Transgenic animals, which can be generated in high numbers in fish, help solve this problem, but there are still a limited number of studies that have specifically perturbed the TME in this setting. This can be a major area for growth.

### Metastasis and the TME

There are several reasons why the zebrafish is a novel model for understanding the contribution of the TME to metastasis. It is of interest that xenograft and allograft models readily metastasize in the zebrafish, yet transgenic tumors do so at a much lower rate. The reasons for this are likely both technical and biological. Mosaic transgenics make it difficult to discern multifocal primaries versus metastases. More importantly, most of the available zebrafish transgenics rely on one to three oncogenes/tumor suppressors, whereas metastasis often requires either more complex genetics and/or epigenetic evolution. It is likely that newer multiplexed transgenics will develop metastasis spontaneously, as has been seen in the mouse. Despite these caveats, as mentioned above, multi-scale imaging of the entire metastatic cascade is possible in zebrafish (i.e. from single cells to whole animals). For example, both intravasation and extravasation require cells to traverse the endothelial cell layer of blood vessels, which can be readily imaged using transgenic reporters such as the *fli1a*-GFP line (Lawson and Weinstein, 2002). Survival in circulation is tightly linked to the redox state of the tumor cell, which is influenced by nutrient availability within the TME, and reporters for NAD/NADH or redox state could be used to assess the mechanisms of success or failure of extravasation (Hung et al., 2011; Zhao et al., 2016). Outgrowth is often regulated by interaction with immune cells, which can be imaged using the *rag2*-GFP (Langenau et al., 2004) or *mpeg1*-GFP (Ellett et al., 2011) transgenic lines. In some organs, survival and outgrowth are mediated by local factors in that new TME, such as the role of plasmin as an endogenous stromal suppressor of brain metastasis, which can be overcome by cancer cell expression of serpins (Valiente et al., 2014). As the number of relevant TME cells continues to be identified through single cell and spatial transcriptomic methods, we can then manipulate specific genes in those TME cells through transgenesis (cDNA or CRISPR) and identify the most relevant mechanisms. Finally, metastasis is increasingly recognized to occur in part through transcriptional, epigenetic, and metabolic reprogramming of the tumor cell, rather than genetic changes in the tumor cell. The zebrafish allows us to rapidly test many combinations of these genes, which are important since each individual gene itself often only has subtle/modest effects, and instead it is the network of genes in the cell that promote overall metastatic phenotypes.

One of the most important, yet poorly understood aspects of metastasis relates to organ tropism. Advances in understanding this question have largely come from transplanting human cells into immunocompromised mice, and then continuously selecting cells that are home to certain organs such as lung or liver. Other approaches have involved using syngeneic mouse tumor cells to avoid immunocompromised conditions (van der Weyden et al., 2017). Despite the power of this approach, transplantation of cells in this manner is largely a readout of engraftment and does not recapitulate all of the canonical steps of this complex process. In this regard, transgenic approaches offer advantages, since they allow metastasis to happen more gradually and likely more closely recapitulates what happens in patients. Using zebrafish models of cancer which can then be coupled with the TME promoter/enhancer reporters can yield new insight into the mechanisms by which those resident TME cells promote or retard each step of metastasis. For example, imaging of liver hepatocytes using a *fabp10*-GFP (Her et al., 2003) could give insight into how these cells play roles in establishment of liver metastases.

### The immune TME

Given the broad interest in immuno-oncology, it is not possible here to review all of the ways in which the zebrafish can contribute to this field. While there have been numerous studies using the fish for this purpose (as discussed above, i.e. Antonio et al., 2015; Britto et al., 2018; Póvoa et al., 2021; van den Berg et al., 2019; Wang et al., 2015), this is still a developing field when compared to mouse or even organoid models. One reason for this is that most human and mouse studies take advantage of antibody cocktails to both analyze and isolate relevant immune cells. In contrast, many if not most of these cell-surface antibodies do not work in the fish system. But in the past few years, there has been a concerted effort to identify the repertoire of immune cells in the zebrafish (Moore et al., 2016), and several new and exciting transgenic lines have been developed that allow us to circumvent the need for antibodies. Transgenes marking CD4+ T-regulatory cells (Kasheta et al., 2017), T cells (Langenau et al., 2003), B-cells (Burroughs-Garcia et al., 2019; Liu et al., 2017), macrophages, and neutrophils (Gray et al., 2011) are all widely available and could easily be crossed into the existing cancer models in fish or used to make de novo tumors with techniques such as tumor cell transplantation, transgenesis, or TEAZ. This would open up exciting new possibilities in applying the zebrafish not only to understanding immunobiology in cancer, but using it for drug or genetic screens to find new immunotherapies that would be hard to screen for in mice. Examples of these approaches are starting to emerge, such as imaging of T-cell-based therapies with single-cell resolution (Yan et al., 2021). Despite this potential, it still remains unknown whether the repertoire of zebrafish immune cells responds to things like neoantigens in the same way as mammalian models. For screening and imaging purposes, the fish may offer some advantages over mice, but validation of their human relevance will require validation in mammalian tissues.

### Drug therapy and the TME

Advances in both targeted and immunotherapy approaches have dramatically altered our approach to treating cancer. Despite these improvements, the majority of patients with metastatic disease die from their disease, which is primarily due to drug resistance. In the case of molecularly targeted therapy, this is in part due to the influence of the TME. For example, a mainstay of therapy for advanced melanoma is combination therapy with BRAF/MEK inhibitors, but resistance develops rapidly to this combination. Several TME-specific mechanisms have been shown to contribute to drug resistance in cancer. Stromal secretion of HGF, the ligand for the c-MET receptor, is an important cause of such resistance (Straussman et al., 2012). The TME can also regulate tumor growth via complex interplay amidst TME cell types. A recent study showed that aged fibroblasts in the TME secrete fatty acids which can be taken up by both the tumor cells and immune cells in the TME, both of which contribute to resistance to BRAF/MEK inhibitors (Alicea et al., 2020). Blockade of this fatty acid uptake via inhibition of the fatty acid transporter FATP overcame this resistance, highlighting how targeting the TME offers new therapeutic interventions. Thus, a coordinated interplay between multiple TME factors and cell types induces resistance.

The zebrafish as a model offers a real opportunity to dissect these intricate mechanisms in vivo. For example, gene expression profiling of the TME around resistant tumor cells could reveal candidates which can then be genetically tested in a systematic fashion using the promoter constructs described above. Furthermore, high throughput chemical screens (Kaufman et al., 2009) can be used to identify novel approaches to targeting tumor-TME interactions and genetic dependencies underlying the acquisition of resistance. The primary advantage of the zebrafish in drug discovery is the ability to do high throughput chemical screens in a living whole-animal system. Because multiple zebrafish embryo/larvae can fit in a 96-well dish, embryos and larvae are especially apt for chemical screening of hundreds to thousands of compounds at a time. Compounds are absorbed through the water by the gills of the embryo, making drug delivery technically very simple (Letrado et al., 2018; Zhang et al., 2015). Examples in which the zebrafish have led to clinically relevant human drug trials are beginning to increase. Recent reviews (Cully, 2019; Patton et al., 2021) highlight numerous examples of drugs that were initially discovered in zebrafish models but then went to humans. For example, ProHema, a PGE2 derivative, was shown to promote HSC expansion in fish (North et al., 2007), then in human cord blood transplants (Goessling et al., 2011), and is now being tested in a Phase II trial for the treatment of leukemia and graft versus host disease. Adult PDX and xenograft models of rhabdomyosarcoma in zebrafish have led to the identification of olaparib plus temozolomide (Yan et al., 2019) now being tested in a Phase I clinical trial.

Despite the immense potential of chemical screening in zebrafish, most of these approaches have targeted the tumor cell itself rather than the TME specifically. We speculate that this is primarily due to the adult onset of most tumor models, which makes unbiased chemical screens more difficult in adults when compared to treating embryos. But this has been rapidly improving, in which adults can be drug treated through multiple routes, such as dissolving drug in water, oral gavage (Dang et al., 2016), food pellets (Lu and Patton, 2022), intratumoral injection, retro-orbital injection (Pugach et al., 2009), and intraperitoneal injection, but this is still not as high throughput as the embryonic assay.

While many bioactive compounds have conserved activity between fish and humans, there are some cases of drugs demonstrating species specificity to humans or fish (e.g. differences in ligand specificity for estrogens and anti-estrogens), there are other cases where zebrafish more effectively predict drug toxicity than in mouse models (thalidomide) (Asnake et al., 2019; Letrado et al., 2018; Patton et al., 2021). While TME-centered chemicals will be discovered through screens in zebrafish, it will take mammalian models to turn these into drugs with favorable pharmacokinetic/pharmacodynamic properties.

### Conclusions

The cancer field is rapidly moving away from the idea of tumor cells acting in cell autonomous ways. Both the TME and host response play instructive roles in shaping tumor cell behavior and affecting therapy and patient outcome. Studying this complex ‘whole body’ physiology cannot be accomplished by any one model, whether that is human, mice, fish, flies, or other emerging model organisms. Here we present a rationale for using the zebrafish as a powerful system to functionally interrogate the TME using imaging, genetic, and screening approaches. We can capitalize on many existing cell-type-specific promoters to leverage new and exciting non-germline transgenic approaches and make this more rapid and highly scalable. This new understanding will allow us to ultimately identify new therapeutic vulnerabilities aimed at interrupting the tumor/TME crosstalk.

## Additional files

Supplementary file 1. A listing of the promoter/enhancer elements or transgenic constructs in zebrafish that have been used to mark cell types, organelles or cellular functions.

## References

[bib1] Abe G, Suster ML, Kawakami K (2011). Tol2-mediated transgenesis, gene trapping, enhancer trapping, and the gal4-UAS system. Methods in Cell Biology.

[bib2] Ablain J, Durand EM, Yang S, Zhou Y, Zon LI (2015). A CRISPR/Cas9 vector system for tissue-specific gene disruption in zebrafish. Developmental Cell.

[bib3] Ablain J, Zon LI (2016). Tissue-Specific gene targeting using CRISPR/Cas9. Methods in Cell Biology.

[bib4] Ablain J, Xu M, Rothschild H, Jordan RC, Mito JK, Daniels BH, Bell CF, Joseph NM, Wu H, Bastian BC, Zon LI, Yeh I (2018). Human tumor genomics and zebrafish modeling identify SPRED1 loss as a driver of mucosal melanoma. Science.

[bib5] Akerberg AA, Stewart S, Stankunas K (2014). Spatial and temporal control of transgene expression in zebrafish. PLOS ONE.

[bib6] Albrengues J, Bertero T, Grasset E, Bonan S, Maiel M, Bourget I, Philippe C, Herraiz Serrano C, Benamar S, Croce O, Sanz-Moreno V, Meneguzzi G, Feral CC, Cristofari G, Gaggioli C (2015). Epigenetic switch drives the conversion of fibroblasts into proinvasive cancer-associated fibroblasts. Nature Communications.

[bib7] Alicea GM, Rebecca VW, Goldman AR, Fane ME, Douglass SM, Behera R, Webster MR, Kugel CH, Ecker BL, Caino MC, Kossenkov AV, Tang HY, Frederick DT, Flaherty KT, Xu X, Liu Q, Gabrilovich DI, Herlyn M, Blair IA, Schug ZT, Speicher DW, Weeraratna AT (2020). Changes in aged fibroblast lipid metabolism induce age-dependent melanoma cell resistance to targeted therapy via the fatty acid transporter FATP2. Cancer Discovery.

[bib8] Antonio N, Bønnelykke-Behrndtz ML, Ward LC, Collin J, Christensen IJ, Steiniche T, Schmidt H, Feng Y, Martin P (2015). The wound inflammatory response exacerbates growth of pre-neoplastic cells and progression to cancer. The EMBO Journal.

[bib9] Asnake S, Modig C, Olsson PE (2019). Species differences in ligand interaction and activation of estrogen receptors in fish and human. The Journal of Steroid Biochemistry and Molecular Biology.

[bib10] Astin JW, Jamieson SMF, Eng TCY, Flores MV, Misa JP, Chien A, Crosier KE, Crosier PS (2014). An in vivo antilymphatic screen in zebrafish identifies novel inhibitors of mammalian lymphangiogenesis and lymphatic-mediated metastasis. Molecular Cancer Therapeutics.

[bib11] Auer TO, Duroure K, De Cian A, Concordet JP, Del Bene F (2014). Highly efficient CRISPR/cas9-mediated knock-in in zebrafish by homology-independent DNA repair. Genome Research.

[bib12] Balciunas D, Davidson AE, Sivasubbu S, Hermanson SB, Welle Z, Ekker SC (2004). Enhancer trapping in zebrafish using the sleeping beauty transposon. BMC Genomics.

[bib13] Baranasic D, Hörtenhuber M, Balwierz PJ, Zehnder T, Mukarram AK, Nepal C, Várnai C, Hadzhiev Y, Jimenez-Gonzalez A, Li N, Wragg J, D’Orazio FM, Relic D, Pachkov M, Díaz N, Hernández-Rodríguez B, Chen Z, Stoiber M, Dong M, Stevens I, Ross SE, Eagle A, Martin R, Obasaju O, Rastegar S, McGarvey AC, Kopp W, Chambers E, Wang D, Kim HR, Acemel RD, Naranjo S, Łapiński M, Chong V, Mathavan S, Peers B, Sauka-Spengler T, Vingron M, Carninci P, Ohler U, Lacadie SA, Burgess SM, Winata C, van Eeden F, Vaquerizas JM, Gómez-Skarmeta JL, Onichtchouk D, Brown BJ, Bogdanovic O, van Nimwegen E, Westerfield M, Wardle FC, Daub CO, Lenhard B, Müller F (2022). Multiomic atlas with functional stratification and developmental dynamics of zebrafish cis-regulatory elements. Nature Genetics.

[bib14] Barone V, Lang M, Krens SFG, Pradhan SJ, Shamipour S, Sako K, Sikora M, Guet CC, Heisenberg CP (2017). An effective feedback loop between cell-cell contact duration and morphogen signaling determines cell fate. Developmental Cell.

[bib15] Benaduce AP, Batista D, Grilo G, Jorge K, Cardero D, Milikowski C, Kos L (2014). Novel UV-induced melanoma mouse model dependent on endothelin3 signaling. Pigment Cell & Melanoma Research.

[bib16] Breznik B, Motaln H, Vittori M, Rotter A, Lah Turnšek T (2017). Mesenchymal stem cells differentially affect the invasion of distinct glioblastoma cell lines. Oncotarget.

[bib17] Britto DD, Wyroba B, Chen W, Lockwood RA, Tran KB, Shepherd PR, Hall CJ, Crosier KE, Crosier PS, Astin JW (2018). Macrophages enhance vegfa-driven angiogenesis in an embryonic zebrafish tumour xenograft model. Disease Models & Mechanisms.

[bib18] Burger A, Lindsay H, Felker A, Hess C, Anders C, Chiavacci E, Zaugg J, Weber LM, Catena R, Jinek M, Robinson MD, Mosimann C (2016). Maximizing mutagenesis with solubilized CRISPR-cas9 ribonucleoprotein complexes. Development.

[bib19] Burroughs-Garcia J, Hasan A, Park G, Borga C, Frazer JK (2019). Isolating malignant and non-malignant B cells from lck:egfp zebrafish. Journal of Visualized Experiments.

[bib20] Cadiz L, Jonz MG (2020). A comparative perspective on lung and gill regeneration. The Journal of Experimental Biology.

[bib21] Callahan SJ, Tepan S, Zhang YM, Lindsay H, Burger A, Campbell NR, Kim IS, Hollmann TJ, Studer L, Mosimann C, White RM (2018). Cancer modeling by transgene electroporation in adult zebrafish (TEAZ). Disease Models & Mechanisms.

[bib22] Campbell NR, Rao A, Hunter MV, Sznurkowska MK, Briker L, Zhang M, Baron M, Heilmann S, Deforet M, Kenny C, Ferretti LP, Huang TH, Perlee S, Garg M, Nsengimana J, Saini M, Montal E, Tagore M, Newton-Bishop J, Middleton MR, Corrie P, Adams DJ, Rabbie R, Aceto N, Levesque MP, Cornell RA, Yanai I, Xavier JB, White RM (2021). Cooperation between melanoma cell states promotes metastasis through heterotypic cluster formation. Developmental Cell.

[bib23] Cavey M, Lecuit T (2009). Molecular bases of cell-cell junctions stability and dynamics. Cold Spring Harbor Perspectives in Biology.

[bib24] Ceol CJ, Houvras Y, Jane-Valbuena J, Bilodeau S, Orlando DA, Battisti V, Fritsch L, Lin WM, Hollmann TJ, Ferré F, Bourque C, Burke CJ, Turner L, Uong A, Johnson LA, Beroukhim R, Mermel CH, Loda M, Ait-Si-Ali S, Garraway LA, Young RA, Zon LI (2011). The histone methyltransferase SETDB1 is recurrently amplified in melanoma and accelerates its onset. Nature.

[bib25] Chaffer CL, Weinberg RA (2011). A perspective on cancer cell metastasis. Science.

[bib26] Chan IS, Knútsdóttir H, Ramakrishnan G, Padmanaban V, Warrier M, Ramirez JC, Dunworth M, Zhang H, Jaffee EM, Bader JS, Ewald AJ (2020). Cancer cells educate natural killer cells to a metastasis-promoting cell state. The Journal of Cell Biology.

[bib27] Cully M (2019). Zebrafish earn their drug discovery stripes. Nature Reviews. Drug Discovery.

[bib28] Curado S, Stainier DYR, Anderson RM (2008). Nitroreductase-mediated cell/tissue ablation in zebrafish: a spatially and temporally controlled ablation method with applications in developmental and regeneration studies. Nature Protocols.

[bib29] Dang M, Henderson RE, Garraway LA, Zon LI (2016). Long-term drug administration in the adult zebrafish using oral gavage for cancer preclinical studies. Disease Models & Mechanisms.

[bib30] Dankort D, Curley DP, Cartlidge RA, Nelson B, Karnezis AN, Damsky WE, You MJ, DePinho RA, McMahon M, Bosenberg M (2009). Braf(V600E) cooperates with pten loss to induce metastatic melanoma. Nature Genetics.

[bib31] Dhomen N, Reis-Filho JS, da Rocha Dias S, Hayward R, Savage K, Delmas V, Larue L, Pritchard C, Marais R (2009). Oncogenic braf induces melanocyte senescence and melanoma in mice. Cancer Cell.

[bib32] Dumortier JG, Martin S, Meyer D, Rosa FM, David NB (2012). Collective mesendoderm migration relies on an intrinsic directionality signal transmitted through cell contacts. PNAS.

[bib33] Eisenhoffer GT, Slattum G, Ruiz OE, Otsuna H, Bryan CD, Lopez J, Wagner DS, Bonkowsky JL, Chien CB, Dorsky RI, Rosenblatt J (2017). A toolbox to study epidermal cell types in zebrafish. Journal of Cell Science.

[bib34] Ellett F, Pase L, Hayman JW, Andrianopoulos A, Lieschke GJ (2011). Mpeg1 promoter transgenes direct macrophage-lineage expression in zebrafish. Blood.

[bib35] Emelyanov A, Parinov S (2008). Mifepristone-inducible lexpr system to drive and control gene expression in transgenic zebrafish. Developmental Biology.

[bib36] Esengil H, Chang V, Mich JK, Chen JK (2007). Small-molecule regulation of zebrafish gene expression. Nature Chemical Biology.

[bib37] Farrell JA, Wang Y, Riesenfeld SJ, Shekhar K, Regev A, Schier AF (2018). Single-cell reconstruction of developmental trajectories during zebrafish embryogenesis. Science.

[bib38] Fazio M, Ablain J, Chuan Y, Langenau DM, Zon LI (2020). Zebrafish patient avatars in cancer biology and precision cancer therapy. Nature Reviews. Cancer.

[bib39] Feng Y, Santoriello C, Mione M, Hurlstone A, Martin P (2010). Live imaging of innate immune cell sensing of transformed cells in zebrafish larvae: parallels between tumor initiation and wound inflammation. PLOS Biology.

[bib40] Feng Y, Renshaw S, Martin P (2012). Live imaging of tumor initiation in zebrafish larvae reveals a trophic role for leukocyte-derived PGE₂. Current Biology.

[bib41] Fior R, Póvoa V, Mendes RV, Carvalho T, Gomes A, Figueiredo N, Ferreira MG (2017). Single-cell functional and chemosensitive profiling of combinatorial colorectal therapy in zebrafish xenografts. PNAS.

[bib42] Flores MV, Hall CJ, Crosier KE, Crosier PS (2010). Visualization of embryonic lymphangiogenesis advances the use of the zebrafish model for research in cancer and lymphatic pathologies. Developmental Dynamics.

[bib43] Follain G, Osmani N, Azevedo AS, Allio G, Mercier L, Karreman MA, Solecki G, Garcia Leòn MJ, Lefebvre O, Fekonja N, Hille C, Chabannes V, Dollé G, Metivet T, Hovsepian FD, Prudhomme C, Pichot A, Paul N, Carapito R, Bahram S, Ruthensteiner B, Kemmling A, Siemonsen S, Schneider T, Fiehler J, Glatzel M, Winkler F, Schwab Y, Pantel K, Harlepp S, Goetz JG (2018). Hemodynamic forces tune the arrest, adhesion, and extravasation of circulating tumor cells. Developmental Cell.

[bib44] Gilbert LA, Horlbeck MA, Adamson B, Villalta JE, Chen Y, Whitehead EH, Guimaraes C, Panning B, Ploegh HL, Bassik MC, Qi LS, Kampmann M, Weissman JS (2014). Genome-Scale CRISPR-mediated control of gene repression and activation. Cell.

[bib45] Goessling W, Allen RS, Guan X, Jin P, Uchida N, Dovey M, Harris JM, Metzger ME, Bonifacino AC, Stroncek D, Stegner J, Armant M, Schlaeger T, Tisdale JF, Zon LI, Donahue RE, North TE (2011). Prostaglandin E2 enhances human cord blood stem cell xenotransplants and shows long-term safety in preclinical nonhuman primate transplant models. Cell Stem Cell.

[bib46] Golan T, Messer AR, Amitai-Lange A, Melamed Z, Ohana R, Bell RE, Kapitansky O, Lerman G, Greenberger S, Khaled M, Amar N, Albrengues J, Gaggioli C, Gonen P, Tabach Y, Sprinzak D, Shalom-Feuerstein R, Levy C (2015). Interactions of melanoma cells with distal keratinocytes trigger metastasis via notch signaling inhibition of MITF. Molecular Cell.

[bib47] Gómez-Cuadrado L, Tracey N, Ma R, Qian B, Brunton VG (2017). Mouse models of metastasis: progress and prospects. Disease Models & Mechanisms.

[bib48] González F, Zhu Z, Shi ZD, Lelli K, Verma N, Li QV, Huangfu D (2014). An icrispr platform for rapid, multiplexable, and inducible genome editing in human pluripotent stem cells. Cell Stem Cell.

[bib49] Gray C, Loynes CA, Whyte MKB, Crossman DC, Renshaw SA, Chico TJA (2011). Simultaneous intravital imaging of macrophage and neutrophil behaviour during inflammation using a novel transgenic zebrafish. Thrombosis and Haemostasis.

[bib50] Gupta GP, Massagué J (2006). Cancer metastasis: building a framework. Cell.

[bib51] Hans S, Zöller D, Hammer J, Stucke J, Spieß S, Kesavan G, Kroehne V, Eguiguren JS, Ezhkova D, Petzold A, Dahl A, Brand M (2021). Cre-controlled CRISPR mutagenesis provides fast and easy conditional gene inactivation in zebrafish. Nature Communications.

[bib52] Hason M, Bartůněk P (2019). Zebrafish models of cancer-new insights on modeling human cancer in a non-mammalian vertebrate. Genes.

[bib53] Heilmann S, Ratnakumar K, Langdon E, Kansler E, Kim I, Campbell NR, Perry E, McMahon A, Kaufman C, van Rooijen E, Lee W, Iacobuzio-Donahue C, Hynes R, Zon L, Xavier J, White R (2015). A quantitative system for studying metastasis using transparent zebrafish. Cancer Research.

[bib54] Her GM, Chiang CC, Chen WY, Wu JL (2003). In vivo studies of liver-type fatty acid binding protein (L-FABP) gene expression in liver of transgenic zebrafish (*Danio rerio*). FEBS Letters.

[bib55] Hill W, Caswell DR, Swanton C (2021). Capturing cancer evolution using genetically engineered mouse models (gemms). Trends in Cell Biology.

[bib56] Hingorani SR, Petricoin EF, Maitra A, Rajapakse V, King C, Jacobetz MA, Ross S, Conrads TP, Veenstra TD, Hitt BA, Kawaguchi Y, Johann D, Liotta LA, Crawford HC, Putt ME, Jacks T, Wright CVE, Hruban RH, Lowy AM, Tuveson DA (2003). Preinvasive and invasive ductal pancreatic cancer and its early detection in the mouse. Cancer Cell.

[bib57] Hoeppner LH, Sinha S, Wang Y, Bhattacharya R, Dutta S, Gong X, Bedell VM, Suresh S, Chun C, Ramchandran R, Ekker SC, Mukhopadhyay D (2015). RhoC maintains vascular homeostasis by regulating VEGF-induced signaling in endothelial cells. Journal of Cell Science.

[bib58] Hou AJ, Chen LC, Chen YY (2021). Navigating CAR-T cells through the solid-tumour microenvironment. Nature Reviews. Drug Discovery.

[bib59] Hung YP, Albeck JG, Tantama M, Yellen G (2011). Imaging cytosolic NADH-NAD(+) redox state with a genetically encoded fluorescent biosensor. Cell Metabolism.

[bib60] Hunter MV, Moncada R, Weiss JM, Yanai I, White RM (2021). Spatially resolved transcriptomics reveals the architecture of the tumor-microenvironment interface. Nature Communications.

[bib61] Hwang WY, Fu Y, Reyon D, Maeder ML, Tsai SQ, Sander JD, Peterson RT, Yeh JRJ, Joung JK (2013). Efficient genome editing in zebrafish using a CRISPR-Cas system. Nature Biotechnology.

[bib62] Ilander M, Olsson-Strömberg U, Schlums H, Guilhot J, Brück O, Lähteenmäki H, Kasanen T, Koskenvesa P, Söderlund S, Höglund M, Markevärn B, Själander A, Lotfi K, Dreimane A, Lübking A, Holm E, Björeman M, Lehmann S, Stenke L, Ohm L, Gedde-Dahl T, Majeed W, Ehrencrona H, Koskela S, Saussele S, Mahon FX, Porkka K, Hjorth-Hansen H, Bryceson YT, Richter J, Mustjoki S (2017). Increased proportion of mature NK cells is associated with successful imatinib discontinuation in chronic myeloid leukemia. Leukemia.

[bib63] Isiaku AI, Zhang Z, Pazhakh V, Manley HR, Thompson ER, Fox LC, Yerneni S, Blombery P, Lieschke GJ (2021). Transient, flexible gene editing in zebrafish neutrophils and macrophages for determination of cell-autonomous functions. Disease Models & Mechanisms.

[bib64] Jessen JR, Jessen TN, Vogel SS, Lin S (2001). Concurrent expression of recombination activating genes 1 and 2 in zebrafish olfactory sensory neurons. Genesis.

[bib65] Joyce JA, Pollard JW (2009). Microenvironmental regulation of metastasis. Nature Reviews. Cancer.

[bib66] Ju B, Xu Y, He J, Liao J, Yan T, Hew CL, Lam TJ, Gong Z (1999). Faithful expression of green fluorescent protein (GFP) in transgenic zebrafish embryos under control of zebrafish gene promoters. Developmental Genetics.

[bib67] Kasheta M, Painter CA, Moore FE, Lobbardi R, Bryll A, Freiman E, Stachura D, Rogers AB, Houvras Y, Langenau DM, Ceol CJ (2017). Identification and characterization of T reg-like cells in zebrafish. The Journal of Experimental Medicine.

[bib68] Kaufman C.K, White RM, Zon L (2009). Chemical genetic screening in the zebrafish embryo. Nature Protocols.

[bib69] Kaufman CK, Mosimann C, Fan ZP, Yang S, Thomas AJ, Ablain J, Tan JL, Fogley RD, van Rooijen E, Hagedorn EJ, Ciarlo C, White RM, Matos DA, Puller AC, Santoriello C, Liao EC, Young RA, Zon LI (2016). A zebrafish melanoma model reveals emergence of neural crest identity during melanoma initiation. Science.

[bib70] Kaur A, Webster MR, Marchbank K, Behera R, Ndoye A, Kugel CH, Dang VM, Appleton J, O’Connell MP, Cheng P, Valiga AA, Morissette R, McDonnell NB, Ferrucci L, Kossenkov AV, Meeth K, Tang H-Y, Yin X, Wood WH, Lehrmann E, Becker KG, Flaherty KT, Frederick DT, Wargo JA, Cooper ZA, Tetzlaff MT, Hudgens C, Aird KM, Zhang R, Xu X, Liu Q, Bartlett E, Karakousis G, Eroglu Z, Lo RS, Chan M, Menzies AM, Long GV, Johnson DB, Sosman J, Schilling B, Schadendorf D, Speicher DW, Bosenberg M, Ribas A, Weeraratna AT (2016). SFRP2 in the aged microenvironment drives melanoma metastasis and therapy resistance. Nature.

[bib71] Kim IS, Heilmann S, Kansler ER, Zhang Y, Zimmer M, Ratnakumar K, Bowman RL, Simon-Vermot T, Fennell M, Garippa R, Lu L, Lee W, Hollmann T, Xavier JB, White RM (2017). Microenvironment-derived factors driving metastatic plasticity in melanoma. Nature Communications.

[bib72] Knapp JM, Chung P, Simpson JH (2015). Generating customized transgene landing sites and multi-transgene arrays in *Drosophila* using phiC31 integrase. Genetics.

[bib73] Knopf F, Schnabel K, Haase C, Pfeifer K, Anastassiadis K, Weidinger G (2010). Dually inducible teton systems for tissue-specific conditional gene expression in zebrafish. PNAS.

[bib74] Lakshmikanth T, Burke S, Ali TH, Kimpfler S, Ursini F, Ruggeri L, Capanni M, Umansky V, Paschen A, Sucker A, Pende D, Groh V, Biassoni R, Höglund P, Kato M, Shibuya K, Schadendorf D, Anichini A, Ferrone S, Velardi A, Kärre K, Shibuya A, Carbone E, Colucci F (2009). NCRs and DNAM-1 mediate NK cell recognition and lysis of human and mouse melanoma cell lines in vitro and in vivo. The Journal of Clinical Investigation.

[bib75] Lal P, Tanabe H, Suster ML, Ailani D, Kotani Y, Muto A, Itoh M, Iwasaki M, Wada H, Yaksi E, Kawakami K (2018). Identification of a neuronal population in the telencephalon essential for fear conditioning in zebrafish. BMC Biology.

[bib76] Langenau DM, Traver D, Ferrando AA, Kutok JL, Aster JC, Kanki JP, Lin S, Prochownik E, Trede NS, Zon LI, Look AT (2003). Myc-Induced T cell leukemia in transgenic zebrafish. Science.

[bib77] Langenau DM, Ferrando AA, Traver D, Kutok JL, Hezel JPD, Kanki JP, Zon LI, Look AT, Trede NS (2004). In vivo tracking of T cell development, ablation, and engraftment in transgenic zebrafish. PNAS.

[bib78] Langenau DM, Zon LI (2005). The zebrafish: a new model of T-cell and thymic development. Nature Reviews. Immunology.

[bib79] Lawson ND, Weinstein BM (2002). In vivo imaging of embryonic vascular development using transgenic zebrafish. Developmental Biology.

[bib80] Lee RTH, Asharani PV, Carney TJ (2014). Basal keratinocytes contribute to all strata of the adult zebrafish epidermis. PLOS ONE.

[bib81] Leibold J, Ruscetti M, Cao Z, Ho Y-J, Baslan T, Zou M, Abida W, Feucht J, Han T, Barriga FM, Tsanov KM, Zamechek L, Kulick A, Amor C, Tian S, Rybczyk K, Salgado NR, Sánchez-Rivera FJ, Watson PA, de Stanchina E, Wilkinson JE, Dow LE, Abate-Shen C, Sawyers CL, Lowe SW (2020). Somatic tissue engineering in mouse models reveals an actionable role for Wnt pathway alterations in prostate cancer metastasis. Cancer Discovery.

[bib82] Letrado P, de Miguel I, Lamberto I, Díez-Martínez R, Oyarzabal J (2018). Zebrafish: speeding up the cancer drug discovery process. Cancer Research.

[bib83] Lin X, Zhou Q, Lin G, Zhao C, Wen Z (2020). Endoderm-derived myeloid-like metaphocytes in zebrafish gill mediate soluble antigen-induced immunity. Cell Reports.

[bib84] Lister JA, Robertson CP, Lepage T, Johnson SL, Raible DW (1999). Nacre encodes a zebrafish microphthalmia-related protein that regulates neural-crest-derived pigment cell fate. Development.

[bib85] Liu X, Li YS, Shinton SA, Rhodes J, Tang L, Feng H, Jette CA, Look AT, Hayakawa K, Hardy RR (2017). Zebrafish B cell development without a pre-B cell stage, revealed by CD79 fluorescence reporter transgenes. Journal of Immunology.

[bib86] Liu K, Petree C, Requena T, Varshney P, Varshney GK (2019). Expanding the CRISPR toolbox in zebrafish for studying development and disease. Frontiers in Cell and Developmental Biology.

[bib87] Livet J, Weissman TA, Kang H, Draft RW, Lu J, Bennis RA, Sanes JR, Lichtman JW (2007). Transgenic strategies for combinatorial expression of fluorescent proteins in the nervous system. Nature.

[bib88] Longo SK, Guo MG, Ji AL, Khavari PA (2021). Integrating single-cell and spatial transcriptomics to elucidate intercellular tissue dynamics. Nature Reviews. Genetics.

[bib89] Loveless R, Shay C, Teng Y (2020). Unveiling tumor microenvironment interactions using zebrafish models. Frontiers in Molecular Biosciences.

[bib90] Lu J, Ye X, Fan F, Xia L, Bhattacharya R, Bellister S, Tozzi F, Sceusi E, Zhou Y, Tachibana I, Maru DM, Hawke DH, Rak J, Mani SA, Zweidler-McKay P, Ellis LM (2013). Endothelial cells promote the colorectal cancer stem cell phenotype through a soluble form of Jagged-1. Cancer Cell.

[bib91] Lu Y, Patton EE (2022). Long-Term non-invasive drug treatments in adult zebrafish that lead to melanoma drug resistance. Disease Models & Mechanisms.

[bib92] Lumaquin D, Johns E, Montal E, Weiss JM, Ola D, Abuhashem A, White RM (2021). An in vivo reporter for tracking lipid droplet dynamics in transparent zebrafish. eLife.

[bib93] McConnell AM, Noonan HR, Zon LI (2021). Reeling in the zebrafish cancer models. Annual Review of Cancer Biology.

[bib94] Minchin JEN, Rawls JF (2017). A classification system for zebrafish adipose tissues. Disease Models & Mechanisms.

[bib95] Moore FE, Garcia EG, Lobbardi R, Jain E, Tang Q, Moore JC, Cortes M, Molodtsov A, Kasheta M, Luo CC, Garcia AJ, Mylvaganam R, Yoder JA, Blackburn JS, Sadreyev RI, Ceol CJ, North TE, Langenau DM (2016). Single-Cell transcriptional analysis of normal, aberrant, and malignant hematopoiesis in zebrafish. The Journal of Experimental Medicine.

[bib96] Morsut L, Roybal KT, Xiong X, Gordley RM, Coyle SM, Thomson M, Lim WA (2016). Engineering customized cell sensing and response behaviors using synthetic Notch receptors. Cell.

[bib97] Mosimann C, Kaufman CK, Li P, Pugach EK, Tamplin OJ, Zon LI (2011). Ubiquitous transgene expression and cre-based recombination driven by the ubiquitin promoter in zebrafish. Development.

[bib98] Mosimann C, Puller AC, Lawson KL, Tschopp P, Amsterdam A, Zon LI (2013). Site-Directed zebrafish transgenesis into single landing sites with the phiC31 integrase system. Developmental Dynamics.

[bib99] Nakamura M, Srinivasan P, Chavez M, Carter MA, Dominguez AA, La Russa M, Lau MB, Abbott TR, Xu X, Zhao D, Gao Y, Kipniss NH, Smolke CD, Bondy-Denomy J, Qi LS (2019). Anti-CRISPR-mediated control of gene editing and synthetic circuits in eukaryotic cells. Nature Communications.

[bib100] Neal JT, Li X, Zhu J, Giangarra V, Grzeskowiak CL, Ju J, Liu IH, Chiou SH, Salahudeen AA, Smith AR, Deutsch BC, Liao L, Zemek AJ, Zhao F, Karlsson K, Schultz LM, Metzner TJ, Nadauld LD, Tseng YY, Alkhairy S, Oh C, Keskula P, Mendoza-Villanueva D, De La Vega FM, Kunz PL, Liao JC, Leppert JT, Sunwoo JB, Sabatti C, Boehm JS, Hahn WC, Zheng GXY, Davis MM, Kuo CJ (2018). Organoid modeling of the tumor immune microenvironment. Cell.

[bib101] North TE, Goessling W, Walkley CR, Lengerke C, Kopani KR, Lord AM, Weber GJ, Bowman TV, Jang IH, Grosser T, Fitzgerald GA, Daley GQ, Orkin SH, Zon LI (2007). Prostaglandin E2 regulates vertebrate haematopoietic stem cell homeostasis. Nature.

[bib102] Ombrato L, Nolan E, Kurelac I, Mavousian A, Bridgeman VL, Heinze I, Chakravarty P, Horswell S, Gonzalez-Gualda E, Matacchione G, Weston A, Kirkpatrick J, Husain E, Speirs V, Collinson L, Ori A, Lee JH, Malanchi I (2019). Metastatic-niche labelling reveals parenchymal cells with stem features. Nature.

[bib103] Page DM, Wittamer V, Bertrand JY, Lewis KL, Pratt DN, Delgado N, Schale SE, McGue C, Jacobsen BH, Doty A, Pao Y, Yang H, Chi NC, Magor BG, Traver D (2013). An evolutionarily conserved program of B-cell development and activation in zebrafish. Blood.

[bib104] Pan YA, Freundlich T, Weissman TA, Schoppik D, Wang XC, Zimmerman S, Ciruna B, Sanes JR, Lichtman JW, Schier AF (2013). Zebrabow: multispectral cell labeling for cell tracing and lineage analysis in zebrafish. Development.

[bib105] Pascoal S, Salzer B, Scheuringer E, Wenninger-Weinzierl A, Sturtzel C, Holter W, Taschner-Mandl S, Lehner M, Distel M (2020). A preclinical embryonic zebrafish xenograft model to investigate CAR T cells in vivo. Cancers.

[bib106] Pasqual G, Chudnovskiy A, Tas JMJ, Agudelo M, Schweitzer LD, Cui A, Hacohen N, Victora GD (2018). Monitoring T cell-dendritic cell interactions in vivo by intercellular enzymatic labelling. Nature.

[bib107] Pathria P, Louis TL, Varner JA (2019). Targeting tumor-associated macrophages in cancer. Trends in Immunology.

[bib108] Patsialou A, Bravo-Cordero JJ, Wang Y, Entenberg D, Liu H, Clarke M, Condeelis JS (2013). Intravital multiphoton imaging reveals multicellular streaming as a crucial component of in vivo cell migration in human breast tumors. Intravital.

[bib109] Patton EE, Widlund HR, Kutok JL, Kopani KR, Amatruda JF, Murphey RD, Berghmans S, Mayhall EA, Traver D, Fletcher CDM, Aster JC, Granter SR, Look AT, Lee C, Fisher DE, Zon LI (2005). Braf mutations are sufficient to promote nevi formation and cooperate with p53 in the genesis of melanoma. Current Biology.

[bib110] Patton E.E, Zon LI, Langenau DM (2021). Zebrafish disease models in drug discovery: from preclinical modelling to clinical trials. Nature Reviews. Drug Discovery.

[bib111] Paul CD, Bishop K, Devine A, Paine EL, Staunton JR, Thomas SM, Thomas JR, Doyle AD, Miller Jenkins LM, Morgan NY, Sood R, Tanner K (2019). Tissue architectural cues drive organ targeting of tumor cells in zebrafish. Cell Systems.

[bib112] Peinado H, Zhang H, Matei IR, Costa-Silva B, Hoshino A, Rodrigues G, Psaila B, Kaplan RN, Bromberg JF, Kang Y, Bissell MJ, Cox TR, Giaccia AJ, Erler JT, Hiratsuka S, Ghajar CM, Lyden D (2017). Pre-Metastatic niches: organ-specific homes for metastases. Nature Reviews. Cancer.

[bib113] Pekkonen P, Alve S, Balistreri G, Gramolelli S, Tatti-Bugaeva O, Paatero I, Niiranen O, Tuohinto K, Perälä N, Taiwo A, Zinovkina N, Repo P, Icay K, Ivaska J, Saharinen P, Hautaniemi S, Lehti K, Ojala PM (2018). Lymphatic endothelium stimulates melanoma metastasis and invasion via MMP14-dependent Notch3 and β1-integrin activation. eLife.

[bib114] Pisharath H, Rhee JM, Swanson MA, Leach SD, Parsons MJ (2007). Targeted ablation of beta cells in the embryonic zebrafish pancreas using *E. coli* nitroreductase. Mechanisms of Development.

[bib115] Póvoa V, Rebelo de Almeida C, Maia-Gil M, Sobral D, Domingues M, Martinez-Lopez M, de Almeida Fuzeta M, Silva C, Grosso AR, Fior R (2021). Innate immune evasion revealed in a colorectal zebrafish xenograft model. Nature Communications.

[bib116] Premsrirut PK, Dow LE, Kim SY, Camiolo M, Malone CD, Miething C, Scuoppo C, Zuber J, Dickins RA, Kogan SC, Shroyer KR, Sordella R, Hannon GJ, Lowe SW (2011). A rapid and scalable system for studying gene function in mice using conditional RNA interference. Cell.

[bib117] Pugach EK, Li P, White R, Zon L (2009). Retro-orbital injection in adult zebrafish. Journal of Visualized Experiments.

[bib118] Quail DF, Joyce JA (2017). Molecular pathways: deciphering mechanisms of resistance to macrophage-targeted therapies. Clinical Cancer Research.

[bib119] Raj B, Wagner DE, McKenna A, Pandey S, Klein AM, Shendure J, Gagnon JA, Schier AF (2018). Simultaneous single-cell profiling of lineages and cell types in the vertebrate brain. Nature Biotechnology.

[bib120] Rebelo de Almeida C, Mendes RV, Pezzarossa A, Gago J, Carvalho C, Alves A, Nunes V, Brito MJ, Cardoso MJ, Ribeiro J, Cardoso F, Ferreira MG, Fior R (2020). Zebrafish xenografts as a fast screening platform for bevacizumab cancer therapy. Communications Biology.

[bib121] Sacco A, Roccaro AM, Ma D, Shi J, Mishima Y, Moschetta M, Chiarini M, Munshi N, Handin RI, Ghobrial IM (2016). Cancer cell dissemination and homing to the bone marrow in a zebrafish model. Cancer Research.

[bib122] Sahai E, Astsaturov I, Cukierman E, DeNardo DG, Egeblad M, Evans RM, Fearon D, Greten FR, Hingorani SR, Hunter T, Hynes RO, Jain RK, Janowitz T, Jorgensen C, Kimmelman AC, Kolonin MG, Maki RG, Powers RS, Puré E, Ramirez DC, Scherz-Shouval R, Sherman MH, Stewart S, Tlsty TD, Tuveson DA, Watt FM, Weaver V, Weeraratna AT, Werb Z (2020). A framework for advancing our understanding of cancer-associated fibroblasts. Nature Reviews. Cancer.

[bib123] Satou C, Kimura Y, Hirata H, Suster ML, Kawakami K, Higashijima S (2013). Transgenic tools to characterize neuronal properties of discrete populations of zebrafish neurons. Development.

[bib124] Sherman MH, Yu RT, Engle DD, Ding N, Atkins AR, Tiriac H, Collisson EA, Connor F, Van Dyke T, Kozlov S, Martin P, Tseng TW, Dawson DW, Donahue TR, Masamune A, Shimosegawa T, Apte MV, Wilson JS, Ng B, Lau SL, Gunton JE, Wahl GM, Hunter T, Drebin JA, O’Dwyer PJ, Liddle C, Tuveson DA, Downes M, Evans RM (2014). Vitamin D receptor-mediated stromal reprogramming suppresses pancreatitis and enhances pancreatic cancer therapy. Cell.

[bib125] Singleman C, Holtzman NG (2014). Growth and maturation in the zebrafish, *Danio rerio*: a staging tool for teaching and research. Zebrafish.

[bib126] Spanjaard B, Hu B, Mitic N, Olivares-Chauvet P, Janjuha S, Ninov N, Junker JP (2018). Simultaneous lineage tracing and cell-type identification using CRISPR-Cas9-induced genetic scars. Nature Biotechnology.

[bib127] Stoletov K, Montel V, Lester RD, Gonias SL, Klemke R (2007). High-resolution imaging of the dynamic tumor cell vascular interface in transparent zebrafish. PNAS.

[bib128] Straussman R, Morikawa T, Shee K, Barzily-Rokni M, Qian ZR, Du J, Davis A, Mongare MM, Gould J, Frederick DT, Cooper ZA, Chapman PB, Solit DB, Ribas A, Lo RS, Flaherty KT, Ogino S, Wargo JA, Golub TR (2012). Tumour micro-environment elicits innate resistance to Raf inhibitors through HGF secretion. Nature.

[bib129] Suresh S, Rabbie R, Garg M, Lumaquin D, Huang TH, Montal E, Ma Y, Cruz NM, Tang X, Nsengimana J, Newton-Bishop J, Hunter MV, Zhu Y, Chen K, de Stanchina E, Adams DJ, White RM (2022). Identifying the transcriptional drivers of metastasis embedded within localized melanoma. Cancer Discovery.

[bib130] Suster ML, Abe G, Schouw A, Kawakami K (2011). Transposon-Mediated BAC transgenesis in zebrafish. Nature Protocols.

[bib131] Tang R, Murray CW, Linde IL, Kramer NJ, Lyu Z, Tsai MK, Chen LC, Cai H, Gitler AD, Engleman E, Lee W, Winslow MM (2020). A versatile system to record cell-cell interactions. eLife.

[bib132] Toda S, Blauch LR, Tang SKY, Morsut L, Lim WA (2018). Programming self-organizing multicellular structures with synthetic cell-cell signaling. Science.

[bib133] Trede NS, Langenau DM, Traver D, Look AT, Zon LI (2004). The use of zebrafish to understand immunity. Immunity.

[bib134] Valiente M, Obenauf AC, Jin X, Chen Q, Zhang XH-F, Lee DJ, Chaft JE, Kris MG, Huse JT, Brogi E, Massagué J (2014). Serpins promote cancer cell survival and vascular co-option in brain metastasis. Cell.

[bib135] van den Berg MCW, MacCarthy-Morrogh L, Carter D, Morris J, Ribeiro Bravo I, Feng Y, Martin P (2019). Proteolytic and opportunistic breaching of the basement membrane zone by immune cells during tumor initiation. Cell Reports.

[bib136] van der Weyden L, Karp NA, Swiatkowska A, Adams DJ, Speak AO (2017). Genome wide in vivo mouse screen data from studies to assess host regulation of metastatic colonisation. Scientific Data.

[bib137] Venkatesan AM, Vyas R, Gramann AK, Dresser K, Gujja S, Bhatnagar S, Chhangawala S, Gomes CBF, Xi HS, Lian CG, Houvras Y, Edwards YJK, Deng A, Green M, Ceol CJ (2018). Ligand-Activated BMP signaling inhibits cell differentiation and death to promote melanoma. The Journal of Clinical Investigation.

[bib138] Wagner DE, Klein AM (2020). Lineage tracing meets single-cell omics: opportunities and challenges. Nature Reviews. Genetics.

[bib139] Wan H, Korzh S, Li Z, Mudumana SP, Korzh V, Jiang YJ, Lin S, Gong Z (2006). Analyses of pancreas development by generation of GFP transgenic zebrafish using an exocrine pancreas-specific elastasea gene promoter. Experimental Cell Research.

[bib140] Wang J, Cao Z, Zhang XM, Nakamura M, Sun M, Hartman J, Harris RA, Sun Y, Cao Y (2015). Novel mechanism of macrophage-mediated metastasis revealed in a zebrafish model of tumor development. Cancer Research.

[bib141] Wang L, Long J, Chen H, Sun S, Lv K, Li Q, Wang X (2021). Manipulation of focal Wnt activity via synthetic cells in a double-humanized zebrafish model of tumorigenesis. International Journal of Cancer.

[bib142] Wattrus SJ, Zon LI (2018). Stem cell safe harbor: the hematopoietic stem cell niche in zebrafish. Blood Advances.

[bib143] Weiss JM, Hunter MV, Cruz NM, Baggiolini A, Tagore M, Ma Y, Misale S, Marasco M, Simon-Vermot T, Campbell NR, Newell F, Wilmott JS, Johansson PA, Thompson JF, Long GV, Pearson JV, Mann GJ, Scolyer RA, Waddell N, Montal ED, Huang T-H, Jonsson P, Donoghue MTA, Harris CC, Taylor BS, Xu T, Chaligné R, Shliaha PV, Hendrickson R, Jungbluth AA, Lezcano C, Koche R, Studer L, Ariyan CE, Solit DB, Wolchok JD, Merghoub T, Rosen N, Hayward NK, White RM (2022). Anatomic position determines oncogenic specificity in melanoma. Nature.

[bib144] Welker JM, Wierson WA, Almeida MP, Mann CM, Torrie ME, Ming Z, Ekker SC, Clark KJ, Dobbs DL, Essner JJ, McGrail M (2021). GeneWeld: efficient targeted integration directed by short homology in zebrafish. Bio-Protocol.

[bib145] White RM, Sessa A, Burke C, Bowman T, LeBlanc J, Ceol C, Bourque C, Dovey M, Goessling W, Burns CE, Zon LI (2008). Transparent adult zebrafish as a tool for in vivo transplantation analysis. Cell Stem Cell.

[bib146] White R, Rose K, Zon L (2013). Zebrafish cancer: the state of the art and the path forward. Nature Reviews. Cancer.

[bib147] Wierson WA, Welker JM, Almeida MP, Mann CM, Webster DA, Torrie ME, Weiss TJ, Kambakam S, Vollbrecht MK, Lan M, McKeighan KC, Levey J, Ming Z, Wehmeier A, Mikelson CS, Haltom JA, Kwan KM, Chien CB, Balciunas D, Ekker SC, Clark KJ, Webber BR, Moriarity BS, Solin SL, Carlson DF, Dobbs DL, McGrail M, Essner J (2020). Efficient targeted integration directed by short homology in zebrafish and mammalian cells. eLife.

[bib148] Wu RS, Lam II, Clay H, Duong DN, Deo RC, Coughlin SR (2018). A rapid method for directed gene knockout for screening in G0 zebrafish. Developmental Cell.

[bib149] Yan C, Brunson DC, Tang Q, Do D, Iftimia NA, Moore JC, Hayes MN, Welker AM, Garcia EG, Dubash TD, Hong X, Drapkin BJ, Myers DT, Phat S, Volorio A, Marvin DL, Ligorio M, Dershowitz L, McCarthy KM, Karabacak MN, Fletcher JA, Sgroi DC, Iafrate JA, Maheswaran S, Dyson NJ, Haber DA, Rawls JF, Langenau DM (2019). Visualizing engrafted human cancer and therapy responses in immunodeficient zebrafish. Cell.

[bib150] Yan C, Yang Q, Zhang S, Millar DG, Alpert EJ, Do D, Veloso A, Brunson DC, Drapkin BJ, Stanzione M, Scarfò I, Moore JC, Iyer S, Qin Q, Wei Y, McCarthy KM, Rawls JF, Dyson NJ, Cobbold M, Maus MV, Langenau DM (2021). Single-Cell imaging of T cell immunotherapy responses in vivo. The Journal of Experimental Medicine.

[bib151] Zhang F, Qin W, Zhang JP, Hu CQ (2015). Antibiotic toxicity and absorption in zebrafish using liquid chromatography-tandem mass spectrometry. PLOS ONE.

[bib152] Zhang M, Di Martino JS, Bowman RL, Campbell NR, Baksh SC, Simon-Vermot T, Kim IS, Haldeman P, Mondal C, Yong-Gonzales V, Abu-Akeel M, Merghoub T, Jones DR, Zhu XG, Arora A, Ariyan CE, Birsoy K, Wolchok JD, Panageas KS, Hollmann T, Bravo-Cordero JJ, White RM (2018). Adipocyte-Derived lipids mediate melanoma progression via FATP proteins. Cancer Discovery.

[bib153] Zhao Y, Wang A, Zou Y, Su N, Loscalzo J, Yang Y (2016). In vivo monitoring of cellular energy metabolism using sonar, a highly responsive sensor for NAD (+) /NADH redox state. Nature Protocols.

