# Supplementary material for: Shifting the focus of zebrafish toward a model of the tumor microenvironment
Source: eLife. 2022 Dec 20;11:e69703. doi: 10.7554/eLife.69703 (PMC9767465; doi:10.7554/eLife.69703)
Supplement: Supplementary file 1. [file elife-69703-supp1.docx]

Table 2: Cell type specific drivers available in zebrafish

| **Cell Type** | **Subtype or Differentiation State** | **Promoter, Enhancer, or Sensor** | **Citation** |
| --- | --- | --- | --- |
| **DERMATOLOGIC** | | | |
| Melanocyte | General | kita | [(Mort et al., 2015; Santoriello et al., 2010)](http://sciwheel.com:80/work/citation?ids=1035020%2c1055964&pre=&pre=&suf=&suf=&sa=0%2c0) |
| Melanocyte | Melanoblast | mitfa | [(Dorsky et al., 2000; Mort et al., 2015)](http://sciwheel.com:80/work/citation?ids=1035020%2c110855&pre=&pre=&suf=&suf=&sa=0%2c0) |
| Melanocyte | Differentiated melanocyte | tyrp1 | [(Mort et al., 2015; Zou et al., 2006)](http://sciwheel.com:80/work/citation?ids=1035020%2c1648618&pre=&pre=&suf=&suf=&sa=0%2c0) |
| Xanthophore |  | aox5 (formerly referred to as aox3) | [(Eom et al., 2015; Sarah K McMenamin et al., 2014)](http://sciwheel.com:80/work/citation?ids=1130300%2c10531293&pre=&pre=&suf=&suf=&sa=0%2c0) |
| Iridophore |  | tdl358, pnp4a | [(Cal et al., 2019; Patterson and Parichy, 2013)](http://sciwheel.com:80/work/citation?ids=7062547%2c3972580&pre=&pre=&suf=&suf=&sa=0%2c0) |
| Neural crest |  | crestin, sox10 | [(Carney et al., 2006; Kaufman et al., 2016; White et al., 2011)](http://sciwheel.com:80/work/citation?ids=1192845%2c239918%2c232111&pre=&pre=&pre=&suf=&suf=&suf=&sa=0%2c0%2c0) |
| Keratinocyte | Basal | krtt1c19e, tp63 | [(Eisenhoffer et al., 2017; Lee et al., 2014)](http://sciwheel.com:80/work/citation?ids=5620754%2c1437146&pre=&pre=&suf=&suf=&sa=0%2c0) |
| Keratinocyte | Superficial | krt4 (formerly referred to as krt8) | [(Gong et al., 2002; Ju et al., 1999)](http://sciwheel.com:80/work/citation?ids=10399152%2c1082189&pre=&pre=&suf=&suf=&sa=0%2c0) |
| Adipocyte |  | plin2 | [(Zhang et al., 2018)](http://sciwheel.com:80/work/citation?ids=5518626&pre=&suf=&sa=0) |
| Langerhans cell |  | cd4-1 and mpeg1 double positive | [(Lin et al., 2019)](http://sciwheel.com:80/work/citation?ids=9745802&pre=&suf=&sa=0) |
| Metaphocyte |  | cldnh and mpeg1 double positive | [(Lin et al., 2019)](http://sciwheel.com:80/work/citation?ids=9745802&pre=&suf=&sa=0) |
| **HEMATOPOIETIC AND IMMUNE** | | | |
| B-cell |  | cd79 | [(Liu et al., 2017)](http://sciwheel.com:80/work/citation?ids=10531294&pre=&suf=&sa=0) |
| T-cell | Pan-marker | lck, rag2 | [(Jessen et al., 2001; Langenau and Zon, 2005; Langenau et al., 2004)](http://sciwheel.com:80/work/citation?ids=2284989%2c858995%2c9420971&pre=&pre=&pre=&suf=&suf=&suf=&sa=0%2c0%2c0) |
| T-cell | CD4 | cd4-1 | [(Dee et al., 2016)](http://sciwheel.com:80/work/citation?ids=3261279&pre=&suf=&sa=0) |
| T-cell | CD8 | ? |  |
| T-cell | T-reg | foxp3a | [(Hui et al., 2017)](http://sciwheel.com:80/work/citation?ids=4634337&pre=&suf=&sa=0) |
| Neutrophils and Macrophages |  | lyz (also referred to as lysC), myd88 | [(Hall et al., 2009, 2007)](http://sciwheel.com:80/work/citation?ids=1056485%2c1082194&pre=&pre=&suf=&suf=&sa=0%2c0) |
| Neutrophil |  | mpx (also referred to as mpo) | [(Renshaw et al., 2006)](http://sciwheel.com:80/work/citation?ids=1245626&pre=&suf=&sa=0) |
| Macrophages | M1 and M2 | mpeg1, mfap4 | [(Ellett et al., 2011; Walton et al., 2015)](http://sciwheel.com:80/work/citation?ids=972715%2c1521756&pre=&pre=&suf=&suf=&sa=0%2c0) |
| Macrophages | M1 only | mpeg1 and tnfa double positive | [(Nguyen‑Chi et al., 2015)](http://sciwheel.com:80/work/citation?ids=460292&pre=&suf=&sa=0) |
| Macrophages | Activation | mpeg1 and irg1 double positive | [(Sanderson et al., 2015)](http://sciwheel.com:80/work/citation?ids=10531296&pre=&suf=&sa=0) |
| Macrophages and dendritic cells |  | mhc2dab and cd45 double positive | [(Wittamer et al., 2011)](http://sciwheel.com:80/work/citation?ids=66797&pre=&suf=&sa=0) |
| Eosinophils |  | gata2 | [(Balla et al., 2010)](http://sciwheel.com:80/work/citation?ids=1009093&pre=&suf=&sa=0) |
| NK cell |  | ? |  |
| Megakaryocytes |  | mpl | [(Lin et al., 2017)](http://sciwheel.com:80/work/citation?ids=10531297&pre=&suf=&sa=0) |
| Erythrocytes |  | gata1 | [(Long et al., 1997)](http://sciwheel.com:80/work/citation?ids=675986&pre=&suf=&sa=0) |
| Haematopoietic stem and progenitor cell |  | runx, scl-alpha | [(Tamplin et al., 2015; Zhen et al., 2013)](http://sciwheel.com:80/work/citation?ids=676249%2c463572&pre=&pre=&suf=&suf=&sa=0%2c0) |
| **VASCULAR** | | | |
| Vascular smooth muscle cells and pericytes |  | acta2, abcc9, pdgfrb | [(Ando et al., 2019, 2016; Thomas R. Whitesell et al., 2014)](http://sciwheel.com:80/work/citation?ids=10531298%2c11155972%2c4932707&pre=&pre=&pre=&suf=&suf=&suf=&sa=0%2c0%2c0) |
| Vascular endothelium |  | fli1a, flk1 (also referred to as kdrl), cdh5 (also referred to as VE-cadherin), dll4, flt4, scl-beta, tie2 | [(Chávez et al., 2016; Cross et al., 2003; Hogan et al., 2009; Lawson and Weinstein, 2002; Lenard et al., 2013; Motoike et al., 2000; Sacilotto et al., 2013; Stratman et al., 2017; Zhen et al., 2013)](http://sciwheel.com:80/work/citation?ids=2772592%2c1344360%2c675953%2c859251%2c54066%2c675834%2c463572%2c675697%2c65471&pre=&pre=&pre=&pre=&pre=&pre=&pre=&pre=&pre=&suf=&suf=&suf=&suf=&suf=&suf=&suf=&suf=&suf=&sa=0%2c0%2c0%2c0%2c0%2c0%2c0%2c0%2c0) |
| Arterial endothelium |  | efnb2a, nkx2.3, flt1 | [(Chávez et al., 2016; Choe et al., 2013; Hogan et al., 2009; Swift et al., 2014)](http://sciwheel.com:80/work/citation?ids=1344360%2c10531299%2c10398028%2c675834&pre=&pre=&pre=&pre=&suf=&suf=&suf=&suf=&sa=0%2c0%2c0%2c0) |
| Angioblast precursors |  | flk1 | [(Chávez et al., 2016; Cross et al., 2003)](http://sciwheel.com:80/work/citation?ids=1344360%2c675697&pre=&pre=&suf=&suf=&sa=0%2c0) |
| Venous and Lymphatic endothelium  Perivascular fibroblasts |  | prox1a, lyve1b, mrc1a, flt4  pdgfra, col1a2 | [(Jung et al., 2017; Okuda et al., 2012; van Impel et al., 2014)](http://sciwheel.com:80/work/citation?ids=6764247%2c4273277%2c230840&pre=&pre=&pre=&suf=&suf=&suf=&sa=0%2c0%2c0)  [(Rajan et al., 2020; Wang et al., 2020)](http://sciwheel.com:80/work/citation?ids=9769913%2c10116210&pre=&pre=&suf=&suf=&sa=0%2c0) |
| **NEURAL** | | | |
| Neuron | General | alpha1-tubulin, elavl3 (also known as HuC), REx2 | [(Bergeron et al., 2012; Gulati‑Leekha and Goldman, 2006; Park et al., 2000; Satou et al., 2013)](http://sciwheel.com:80/work/citation?ids=230809%2c759154%2c1698%2c2773060&pre=&pre=&pre=&pre=&suf=&suf=&suf=&suf=&sa=0%2c0%2c0%2c0) |
| Neuron | Glutamate | vglut2a | [(Satou et al., 2013, 2012)](http://sciwheel.com:80/work/citation?ids=230809%2c2773097&pre=&pre=&suf=&suf=&sa=0%2c0) |
| Neuron | Glycine | glyt2 | [(McLean et al., 2007; Satou et al., 2020, 2013)](http://sciwheel.com:80/work/citation?ids=230809%2c8427935%2c883941&pre=&pre=&pre=&suf=&suf=&suf=&sa=0%2c0%2c0) |
| Neuron | GABA | gad1b, dlx5/6 | [(Satou et al., 2013; Yu et al., 2011)](http://sciwheel.com:80/work/citation?ids=230809%2c2773090&pre=&pre=&suf=&suf=&sa=0%2c0) |
| Neuron | Dopamine | th1, dat, vmat2 | [(Outeiro, 2017; Wen et al., 2008; Xi et al., 2011)](http://sciwheel.com:80/work/citation?ids=10531300%2c2773159%2c8539652&pre=&pre=&pre=&suf=&suf=&suf=&sa=0%2c0%2c0) |
| Neuron | Serotonin | pet1 | [(Lillesaar et al., 2009)](http://sciwheel.com:80/work/citation?ids=627582&pre=&suf=&sa=0) |
| Neuron | Sensory | isl1, isl2b, crest3, trpa1a, trka, trkb, trkc, p2x3-2 | [(Palanca et al., 2013; Pittman et al., 2008; Uemura et al., 2005)](http://sciwheel.com:80/work/citation?ids=696165%2c3050700%2c78991&pre=&pre=&pre=&suf=&suf=&suf=&sa=0%2c0%2c0) |
| Neuron | Interneuron | alx (also known as chx10), eng1b | [(Higashijima et al., 2004; Kimura et al., 2006)](http://sciwheel.com:80/work/citation?ids=3464246%2c883910&pre=&pre=&suf=&suf=&sa=0%2c0) |
| Neuron | Motor | mnx1 | [(Formella et al., 2018; Zelenchuk and Brusés, 2011)](http://sciwheel.com:80/work/citation?ids=8147902%2c2150&pre=&pre=&suf=&suf=&sa=0%2c0) |
| Neuron | Specific to a neuroanatomical region | atoh1a, neurog1, gsx1, gsx2, dbx1a, dbx1b, dbx2, dmrt3a, ptf1a, spx1, adcyap1a, bcl6a, cabp7b, cadm4, chata, cart2, chodl, cxcr4b, dbh, dmbx1b, drd1b, drd2a, foxb1a, foxb1b, gad2, galn, hcrt, id2b, j1129, kctd15, lhx2b, lhx9, lrrtm4l2, nos1, opn4b, otbpa, otx1b, oxtl, pcp4a, penka, phox2b, rorb, slc6a4a, slit1a, sp5l, sst3, tac1, tbx20, tmem200a, tph2 | [(Burgess et al., 2009; Coffey et al., 2013; Förster et al., 2017; Fujimoto et al., 2011; Heffer et al., 2017; Kani et al., 2010; Kim et al., 2019; Marquart et al., 2015; Nechiporuk et al., 2007; Satou et al., 2013, 2012; Singh et al., 2015; Yokogawa et al., 2012)](http://sciwheel.com:80/work/citation?ids=8402667%2c230809%2c473292%2c2773097%2c3948022%2c1234294%2c1344662%2c1056555%2c10531301%2c4693600%2c3906539%2c1234292%2c2773106&pre=&pre=&pre=&pre=&pre=&pre=&pre=&pre=&pre=&pre=&pre=&pre=&pre=&suf=&suf=&suf=&suf=&suf=&suf=&suf=&suf=&suf=&suf=&suf=&suf=&suf=&sa=0%2c0%2c0%2c0%2c0%2c0%2c0%2c0%2c0%2c0%2c0%2c0%2c0) |
| Astrocytes |  | gfap | [(Bernardos and Raymond, 2006)](http://sciwheel.com:80/work/citation?ids=458&pre=&suf=&sa=0) |
| Radial glia |  | her4.3, myl7 | [(Duncan et al., 2016; Yeo et al., 2007)](http://sciwheel.com:80/work/citation?ids=3157090%2c2702773&pre=&pre=&suf=&suf=&sa=0%2c0) |
| Oligodendrocyte | Oligodendrocyte lineage cells | olig1, olig2, plp | [(D’Rozario et al., 2017; Park et al., 2007; Preston and Macklin, 2015; Schebesta and Serluca, 2009; Shin et al., 2003; Yoshida and Macklin, 2005)](http://sciwheel.com:80/work/citation?ids=3303973%2c886544%2c10531302%2c2702659%2c5782377%2c10531303&pre=&pre=&pre=&pre=&pre=&pre=&suf=&suf=&suf=&suf=&suf=&suf=&sa=0%2c0%2c0%2c0%2c0%2c0) |
| Oligodendrocyte | Precursor and early myelinating | olig2, nkx2.2a | [(D’Rozario et al., 2017; Kirby et al., 2006; Park et al., 2007; Preston and Macklin, 2015; Shin et al., 2003)](http://sciwheel.com:80/work/citation?ids=10531303%2c886544%2c3303973%2c2702659%2c126411&pre=&pre=&pre=&pre=&pre=&suf=&suf=&suf=&suf=&suf=&sa=0%2c0%2c0%2c0%2c0) |
| Oligodendrocyte | Mature myelinating | mpz | [(Brösamle and Halpern, 2002; D’Rozario et al., 2017; Preston and Macklin, 2015)](http://sciwheel.com:80/work/citation?ids=886544%2c3303973%2c346345&pre=&pre=&pre=&suf=&suf=&suf=&sa=0%2c0%2c0) |
| Oligodendrocyte and Schwann cell | Myelinating | mbp, cldnk | [(D’Rozario et al., 2017; Early et al., 2018; Münzel et al., 2012)](http://sciwheel.com:80/work/citation?ids=5520292%2c3303973%2c2156764&pre=&pre=&pre=&suf=&suf=&suf=&sa=0%2c0%2c0) |
| Schwann cell | Immature through myelinating | foxd3 | [(D’Rozario et al., 2017; Gilmour et al., 2002)](http://sciwheel.com:80/work/citation?ids=3303973%2c439263&pre=&pre=&suf=&suf=&sa=0%2c0) |
| Microglia |  | mpeg1, coro1a | [(Ellett et al., 2011; Li et al., 2012)](http://sciwheel.com:80/work/citation?ids=972715%2c1055213&pre=&pre=&suf=&suf=&sa=0%2c0) |
| **OPHTHALMOLOGIC** | | | |
| Retinal cell | General | aanat2, ath5, brn3c, arl4ca, cacnb3b, grin2aa, grin2ab | [(Appelbaum et al., 2006; Förster et al., 2017; Masai et al., 2003; Xiao et al., 2005)](http://sciwheel.com:80/work/citation?ids=1007047%2c484404%2c3948022%2c10531304&pre=&pre=&pre=&pre=&suf=&suf=&suf=&suf=&sa=0%2c0%2c0%2c0) |
| Retinal cell | Rod | rho | [(Angueyra and Kindt, 2018; Hamaoka et al., 2002)](http://sciwheel.com:80/work/citation?ids=8813995%2c1223960&pre=&pre=&suf=&suf=&sa=0%2c0) |
| Retinal cell | Cone; all subtypes | -3.2gnat2 | [(Angueyra and Kindt, 2018; Smyth et al., 2008)](http://sciwheel.com:80/work/citation?ids=8813995%2c9444910&pre=&pre=&suf=&suf=&sa=0%2c0) |
| Retinal cell | UV-cone | opn1sws1 | [(Angueyra and Kindt, 2018; Takechi et al., 2003)](http://sciwheel.com:80/work/citation?ids=8813995%2c1223905&pre=&pre=&suf=&suf=&sa=0%2c0) |
| Retinal cell | S-cone | opn1sws2 | [(Angueyra and Kindt, 2018; Takechi et al., 2008)](http://sciwheel.com:80/work/citation?ids=8813995%2c1223716&pre=&pre=&suf=&suf=&sa=0%2c0) |
| Retinal cell | M-cone | opn1mw2 (also referred to as RH2-2) | [(Angueyra and Kindt, 2018; Tsujimura et al., 2007)](http://sciwheel.com:80/work/citation?ids=8813995%2c10531306&pre=&pre=&suf=&suf=&sa=0%2c0) |
| Retinal cell | L-cone | opn1lw1 | [(Angueyra and Kindt, 2018; Tsujimura et al., 2010)](http://sciwheel.com:80/work/citation?ids=8813995%2c3036554&pre=&pre=&suf=&suf=&sa=0%2c0) |
| Retinal cell | Horizontal cell; all subtypes | cx55.5, lhx1a | [(Angueyra and Kindt, 2018; Rao et al., 2017; Weber et al., 2014)](http://sciwheel.com:80/work/citation?ids=8813995%2c4551521%2c4807367&pre=&pre=&pre=&suf=&suf=&suf=&sa=0%2c0%2c0) |
| Retinal cell | Horizontal cell; H1 and/or H4 subtype | cx52.7, cx52.9, cx52.6 | [(Angueyra and Kindt, 2018; Klaassen et al., 2016)](http://sciwheel.com:80/work/citation?ids=8813995%2c5014233&pre=&pre=&suf=&suf=&sa=0%2c0) |
| Retinal cell | Bipolar cell | grm6b, vsx1, nyx | [(Angueyra and Kindt, 2018; Glasauer et al., 2016; Schroeter et al., 2006; Vitorino et al., 2009)](http://sciwheel.com:80/work/citation?ids=8813995%2c3439619%2c1223677%2c1223800&pre=&pre=&pre=&pre=&suf=&suf=&suf=&suf=&sa=0%2c0%2c0%2c0) |
| **MUSKULOSKELETAL** | | | |
| Osteoblasts | Juvenile | col10a1a, runx2 | [(Bergen et al., 2019; Knopf et al., 2011; Mitchell et al., 2013)](http://sciwheel.com:80/work/citation?ids=8329188%2c10531312%2c307209&pre=&pre=&pre=&suf=&suf=&suf=&sa=0%2c0%2c0) |
| Osteoblasts | Mature | osteocalcin, sp7, entpd5a | [(Bergen et al., 2019; Bussmann and Schulte‑Merker, 2011; DeLaurier et al., 2010; Knopf et al., 2011)](http://sciwheel.com:80/work/citation?ids=8329188%2c307209%2c1223664%2c253489&pre=&pre=&pre=&pre=&suf=&suf=&suf=&suf=&sa=0%2c0%2c0%2c0) |
| Osteoclasts |  | ctsk | [(Bergen et al., 2019; Bussmann and Schulte‑Merker, 2011)](http://sciwheel.com:80/work/citation?ids=8329188%2c253489&pre=&pre=&suf=&suf=&sa=0%2c0) |
| Osteoblast-Osteoclast interaction |  | rankl | [(Bergen et al., 2019; To et al., 2012)](http://sciwheel.com:80/work/citation?ids=8329188%2c10531315&pre=&pre=&suf=&suf=&sa=0%2c0) |
| Chondrocytes |  | col2a1a | [(Bergen et al., 2019; Mitchell et al., 2013)](http://sciwheel.com:80/work/citation?ids=8329188%2c10531312&pre=&pre=&suf=&suf=&sa=0%2c0) |
| Skeletal muscle |  | mylz2, tnnc1b, smyhc1 | [(Jackson et al., 2015; Zeng et al., 2005)](http://sciwheel.com:80/work/citation?ids=10531317%2c10531318&pre=&pre=&suf=&suf=&sa=0%2c0) |
| Tendon fibroblast |  | scxa | [(Subramanian et al., 2018)](http://sciwheel.com:80/work/citation?ids=6209413&pre=&suf=&sa=0) |
| Smooth muscle |  | tagln (also referred to as sm22a-b) | [(Seiler et al., 2010)](http://sciwheel.com:80/work/citation?ids=676189&pre=&suf=&sa=0) |
| **CARDIAC** | | | |
| Cardiac muscle |  | cmlc2, my17 | [(Chávez et al., 2016; Kikuchi et al., 2010)](http://sciwheel.com:80/work/citation?ids=1344360%2c307207&pre=&pre=&suf=&suf=&sa=0%2c0) |
| Cardiac muscle | Embryonic or Regenerating | gata4 | [(Chávez et al., 2016; Kikuchi et al., 2010)](http://sciwheel.com:80/work/citation?ids=1344360%2c307207&pre=&pre=&suf=&suf=&sa=0%2c0) |
| Endocardium |  | flk1 (also referred to as kdr and kdrl) | [(Cross et al., 2003)](http://sciwheel.com:80/work/citation?ids=675697&pre=&suf=&sa=0) |
| Cardiac fibroblast |  | col1a2, postnb | [(Sánchez‑Iranzo et al., 2018)](http://sciwheel.com:80/work/citation?ids=10531321&pre=&suf=&sa=0) |
| **GASTROINTESTINAL** | | | |
| Intestinal epithelia |  | fabp2 | [(Her et al., 2004a, 2004b)](http://sciwheel.com:80/work/citation?ids=923792%2c10531323&pre=&pre=&suf=&suf=&sa=0%2c0) |
| Goblet cells |  | muc5.1 | [(Jevtov et al., 2014)](http://sciwheel.com:80/work/citation?ids=1028472&pre=&suf=&sa=0) |
| Enteroendocrine cells |  | neurod1 | [(Ye et al., 2019)](http://sciwheel.com:80/work/citation?ids=7890166&pre=&suf=&sa=0) |
| Hepatocyte |  | fabp10a | [(Her et al., 2003)](http://sciwheel.com:80/work/citation?ids=1782744&pre=&suf=&sa=0) |
| Cholangiocyte |  | krt18 | [(Wilkins et al., 2014)](http://sciwheel.com:80/work/citation?ids=10531324&pre=&suf=&sa=0) |
| Hepatic stellate cells |  | hand2 | [(Yin et al., 2012)](http://sciwheel.com:80/work/citation?ids=8178506&pre=&suf=&sa=0) |
| Pancreatic exocrine and endocrine |  | pdx1 | [(Huang et al., 2001)](http://sciwheel.com:80/work/citation?ids=7695586&pre=&suf=&sa=0) |
| Pancreatic ductal and acinar cells |  | ptf1a | [(Schiavone et al., 2014)](http://sciwheel.com:80/work/citation?ids=49492&pre=&suf=&sa=0) |
| Pancreatic acinar cells |  | elastaseA | [(Wan et al., 2006)](http://sciwheel.com:80/work/citation?ids=5617732&pre=&suf=&sa=0) |
| Pancreatic endocrine cells | General | pax6b | [(Delporte et al., 2008)](http://sciwheel.com:80/work/citation?ids=1078414&pre=&suf=&sa=0) |
| Pancreatic endocrine cells | Beta | insulin | [(Huang et al., 2001)](http://sciwheel.com:80/work/citation?ids=7695586&pre=&suf=&sa=0) |
| Pancreatic endocrine cells | Delta | sst2 | [(G. Wang et al., 2015)](http://sciwheel.com:80/work/citation?ids=589501&pre=&suf=&sa=0) |
| **RENAL** | | | |
| Pronephric ducts |  | cdh17, enpep, pod | [(Hall et al., 2009; Seiler and Pack, 2011; Zhou et al., 2010)](http://sciwheel.com:80/work/citation?ids=923597%2c1056485%2c10531328&pre=&pre=&pre=&suf=&suf=&suf=&sa=0%2c0%2c0) |
| Glomerular podocytes |  | pod | [(Zhou et al., 2010)](http://sciwheel.com:80/work/citation?ids=10531328&pre=&suf=&sa=0) |
| Renal progenitors |  | wt1b | [(Perner et al., 2007; Zhou et al., 2010)](http://sciwheel.com:80/work/citation?ids=10531328%2c307230&pre=&pre=&suf=&suf=&sa=0%2c0) |
| **REPRODUCTIVE** | | | |
| Primordial germ cell |  | nanos3, dnd | [(Dai et al., 2015; Zhou et al., 2018)](http://sciwheel.com:80/work/citation?ids=6727248%2c7662303&pre=&pre=&suf=&suf=&sa=0%2c0) |
| Testes |  | asp, odf, sam | [(Hsu et al., 2010)](http://sciwheel.com:80/work/citation?ids=10164764&pre=&suf=&sa=0) |
| Ovaries |  | vas | [(Lau et al., 2016)](http://sciwheel.com:80/work/citation?ids=8633184&pre=&suf=&sa=0) |
| **ENDOCRINE** | | | |
| Hypothalamus |  | hspGFFDMC76A (enhancer trap), qrfp | [(Chen et al., 2016; Muto et al., 2017)](http://sciwheel.com:80/work/citation?ids=5981408%2c1231371&pre=&pre=&suf=&suf=&sa=0%2c0) |
| Pituitary | Anterior and posterior corticotrophs | pomc | [(Liu et al., 2003)](http://sciwheel.com:80/work/citation?ids=1035130&pre=&suf=&sa=0) |
| Thyroid |  | tg | [(Opitz et al., 2012)](http://sciwheel.com:80/work/citation?ids=4764145&pre=&suf=&sa=0) |
| Parathyroid hormone-secreting cells |  | pth4 | [(Suarez‑Bregua et al., 2017)](http://sciwheel.com:80/work/citation?ids=10531336&pre=&suf=&sa=0) |
| Interrenal organ (Adrenal gland orthologue) | Steroidogenic | star | [(Gutierrez‑Triana et al., 2015)](http://sciwheel.com:80/work/citation?ids=433936&pre=&suf=&sa=0) |
| Interrenal organ (Adrenal gland orthologue) | Chromaffin | dβh | [(Zhu et al., 2012)](http://sciwheel.com:80/work/citation?ids=1036566&pre=&suf=&sa=0) |
| Pineal gland |  | aanat2 | [(Ben‑Moshe Livne et al., 2016)](http://sciwheel.com:80/work/citation?ids=2670869&pre=&suf=&sa=0) |
| Circadian clock |  | per1b | [(Ben‑Moshe Livne et al., 2016; Lahiri et al., 2014)](http://sciwheel.com:80/work/citation?ids=2670869%2c10531339&pre=&pre=&suf=&suf=&sa=0%2c0) |
| **OTHER** | | | |
| Hypoxia |  | phd3 | [(Santhakumar et al., 2012)](http://sciwheel.com:80/work/citation?ids=1476274&pre=&suf=&sa=0) |
| ROS |  | 3EpRE; Grx-RoGFP2 | [(Mourabit et al., 2019)](http://sciwheel.com:80/work/citation?ids=10531340&pre=&suf=&sa=0) |
| Mechanical stress | YAP/TAZ signaling | 4xGTIIC, eef1a1l1 | [(Miesfeld and Link, 2014; Nakajima et al., 2017)](http://sciwheel.com:80/work/citation?ids=3557512%2c10531341&pre=&pre=&suf=&suf=&sa=0%2c0) |
| Hydrogen peroxide |  | HyPerRed, roGFP2-Orp1 | [(Dickmeis et al., 2019; Panieri and Santoro, 2017; Zou et al., 2018)](http://sciwheel.com:80/work/citation?ids=10531343%2c10531344%2c5861733&pre=&pre=&pre=&suf=&suf=&suf=&sa=0%2c0%2c0) |
| pH |  | pHRed | [(Dickmeis et al., 2019; Tantama et al., 2011)](http://sciwheel.com:80/work/citation?ids=10531343%2c129684&pre=&pre=&suf=&suf=&sa=0%2c0) |
| Redox State | Mitochondrial Matrix | Matrix-roGFP | [(Dickmeis et al., 2019; Waypa et al., 2010)](http://sciwheel.com:80/work/citation?ids=10531343%2c691030&pre=&pre=&suf=&suf=&sa=0%2c0) |
| Redox State | Mitochondrial Intermembrane Space | GPD-ro-GFP | [(Dickmeis et al., 2019; Waypa et al., 2010)](http://sciwheel.com:80/work/citation?ids=10531343%2c691030&pre=&pre=&suf=&suf=&sa=0%2c0) |
| ATP/ADP ratio |  | Perceval HR | [(Berg et al., 2009; Dickmeis et al., 2019)](http://sciwheel.com:80/work/citation?ids=10531343%2c227966&pre=&pre=&suf=&suf=&sa=0%2c0) |
| NADH-NAD (+) ratio |  | Peredox | [(Dickmeis et al., 2019; Hung et al., 2011)](http://sciwheel.com:80/work/citation?ids=10531343%2c77392&pre=&pre=&suf=&suf=&sa=0%2c0) |
| NADPH |  | iNap1 | [(Dickmeis et al., 2019; Zou et al., 2018)](http://sciwheel.com:80/work/citation?ids=10531343%2c5861733&pre=&pre=&suf=&suf=&sa=0%2c0) |
| NADH |  | SoNar | [(Dickmeis et al., 2019; Zhao et al., 2016)](http://sciwheel.com:80/work/citation?ids=10531343%2c1779586&pre=&pre=&suf=&suf=&sa=0%2c0) |
| Calcium |  | GCaMP6s | [(Chen et al., 2017; Dickmeis et al., 2019)](http://sciwheel.com:80/work/citation?ids=10531343%2c4920824&pre=&pre=&suf=&suf=&sa=0%2c0) |
| Pyruvate |  | Pyronic FRET | [(Dickmeis et al., 2019; San Martín et al., 2014)](http://sciwheel.com:80/work/citation?ids=10531343%2c2451426&pre=&pre=&suf=&suf=&sa=0%2c0) |
| Lactate |  | Laconic FRET | [(Dickmeis et al., 2019; San Martín et al., 2013)](http://sciwheel.com:80/work/citation?ids=10531343%2c1125379&pre=&pre=&suf=&suf=&sa=0%2c0) |
